# Fetal hyperechoic kidney cohort study and a meta-analysis

**DOI:** 10.3389/fgene.2023.1237912

**Published:** 2023-08-17

**Authors:** Wei Yang, Shujing Zu, Qiu Jin, Yu Liu, Chao Wang, Huimin Shen, Ruijing Wang, Hui Zhang, Meimei Liu

**Affiliations:** ^1^ Department of Obstetrics and Gynecology, The Second Affliliated Hospital of Harbin Medical University, Harbin, China; ^2^ Department of Prenatal Diagnosis, Harbin Red Cross Central Hospital, Harbin, China

**Keywords:** fetus, hyperechoic kidney, prenatal diagnosis, ultrasound, metaanalysis, CMA, CNV-seq

## Abstract

**Objective:** To investigate the positive rate of chromosomal and monogenic etiologies and pregnancy outcomes in fetuses with hyperechoic kidney, and to provide more information for genetic counseling and prognosis evaluation.

**Methods:** We performed a retrospective analysis of 25 cases of hyperechoic kidney diagnosed prenatal in the Second Affiliated Hospital of Harbin Medical University and Harbin Red Cross Central Hospital (January 2017–December 2022). Furthermore, we conducted a meta-analysis of a series of hyperechoic kidneys (HEK) in the literature to assess the incidence of chromosomal and monogenic etiologies, mortality, and pooled odds ratio (OR) estimates of the association between the incidence of these outcomes and other associated ultrasound abnormalities.

**Results:** 25 fetuses of HEK were enrolled in the cohort study, including 14 with isolated hyperechoic kidney (IHK) and 11 with non-isolated hyperechoic kidney (NIHK). Chromosomal aneuploidies were detected in 4 of 20 patients (20%). The detection rate of pathogenic or suspected pathogenic copy number variations (CNVs) was 29% (4/14) for IHK and 37% (4/11) for NIHK. Whole exome sequencing (WES) was performed in 5 fetuses, and pathogenic genes were detected in all of them. The rate of termination of pregnancy was 56% in HEK. 21 studies including 1,178 fetuses were included in the meta-analysis. No case of abnormal chromosome karyotype or (intrauterine death)IUD was reported in fetuses with IHK. In contrast, the positive rate of karyotype in NIHK was 22% and that in HEK was 20%, with the ORs of 0.28 (95% CI 0.16–0.51) and 0.25, (95% CI 0.14–0.44), respectively. The positive rate of (chromosome microarray analysis) CMA in IHK was 59% and that in NIHK was 32%, with the ORs of 1.46 (95% CI 1.33–1.62) and 0.48 (95% CI, 0.28–0.85), respectively. The positive rate of monogenic etiologies in IHK was 31%, with the OR of 0.80 (95% CI 0.25–2.63). In IHK, the termination rate was 21% and neonatal mortality was 13%, with the ORs of 0.26 (95% CI, 0.17–0.40), 1.72 (95% CI, 1.59–1.86), and that in NIHK was 63%, 0.15 (95% CI, 0.10–0.24); 11%, 0.12 (95% CI, 0.06–0.26), respectively. The intrauterine mortality in NIHK group was 2%, with the OR of 0.02 (95% CI, 0.01–0.05). *HNF1B* variant has the highest incidence (26%) in IHK.

**Conclusion:** The positive rate of karyotype was 20% in HEK and 22% in NIHK. The positive rate of CMA was 32% in NIHK and 59% in IHK. The positive rate of IHK monogenic etiologies was 31%. *HNF1B* gene variation is the most common cause of IHK. The overall fetal mortality rate of NIHK is significantly higher than that of IHK. The amount of amniotic fluid, kidney size and the degree of corticomedullary differentiation have a great impact on the prognosis, these indicators should be taken into consideration to guide clinical consultation and decision-making.

## Introduction

Congenital abnormalities of the kidneys and urinary tracts (CAKUT) are one of the most common fetal structural abnormalities, occurring in about 30%–50% of prenatally diagnosed malformations (Hutson et al., 1985; Deng et al., 2022). CAKUT often result in a series of defects in the kidneys and outflow tracts, including the ureters, the bladder, and urethra. The prevalence is estimated at 4–60 per 10,000 births, depending on the registry, with variation due to differences in sample size, method of diagnosis, and ethnic differences between studies (Tain et al., 2016). A subset of CAKUT patients may show only a hyperechoic kidney on fetal ultrasound during pregnancy, showing clear structural abnormalities only in the third trimester or after birth. Fetal hyperechoic kidney (HEK) may be transient in pregnancy, or may be an ultrasound manifestation of CAKUT or some syndromes, HEK are associated with a wide range of etiologies and prognoses. Prenatal counselling and management can be extremely challenging, especially for isolated HEK (Deng et al., 2022).

Hyperechoic kidneys (HEK) are occasionally seen on routine renal ultrasound scan, or with other clinical indications detected on late pregnancy scans, caused by renal abnormalities (Reisman et al., 1991), such as renal dysplasia, fibrosis, interstitial infiltration, tubular/glomerular dilatation, or microcysts (Dias et al., 2014). HEK can be a first indicator of underlying kidney disease and are detected in approximately 1.6 out of 1,000 scans (AM et al., 1983; Yulia et al., 2021), while HEK are almost exclusively pathogenic in childhood the implications of this finding are less clear in the prenatal period (Digby et al., 2021), because of this uncertainty, it may be difficult for families facing such prenatal findings to make informed decisions during pregnancy.

The differential diagnosis of fetal isolated hyperechoic kidney includes autosomal dominant and autosomal recessive polycystic kidney disease (ADPKD and ARPKD) respectively, congenital nephrotic syndrome, renal vein thrombosis and obstructive uropathy (Shuster et al., 2019). In non-isolated fetal hyperechoic kidney, differential diagnoses include aneuploidy, mainly trisomy 13 syndrome, fetal infections such as cytomegalovirus, Meckel-Gruber syndrome and other renal ciliopathies, and other monogenic disorders associated with CAKUT, including overgrowth syndrome, Examples include Beckwith-Wiedemann, Perlman, and Simpson-Golabi-Behmel syndrome (Avni et al., 2012; Shuster et al., 2019).

Traditional karyotyping, such as G-banding, has been the standard method for detecting various chromosomal abnormalities for decades. With the improvement of prenatal diagnosis technology, more and more prenatal fetal abnormalities are diagnosed not only by ultrasound and chromosome karyotype detection, but also by the maturity of CMA and CNV-seq technology, which has greatly improved the detection rate of chromosomal abnormalities. Different genetic diagnosis strategies have also found more gene abnormalities related to HEK. The study (Deng et al., 2022) showed that about 64.29% of fetal HEK were related to genetic factors, and the detection rate of single gene variation was higher than that of chromosome abnormality and copy number variation. And in IHK cases, the positive rate of single gene variants was 50% (4/8). The characteristics of oligohydramnios and fetal kidney size ≥2SD from the mean are associated with poorer outcomes, such as neonatal death (Mashiach et al., 2005), but are often not specific to a single underlying cause. Their absence does not guarantee favorable outcomes (i.e., live birth and child survival without renal impairment) (Digby et al., 2021).

The purpose of this cohort study and meta-analysis was to determine the positive rate of chromosomal and monogenic etiologies, perinatal mortality and related factors in IHK and NIHK fetuses, so that clinicians can determine the prognosis of the fetus and provide accurate counseling for parents better.

## Materials and methods

### Cohort study

We reviewed all 3,995 cases of prenatal diagnosis in the Second Affiliated Hospital of Harbin Medical University and Harbin Red Cross Central Hospital (January 2017 to December 2022), of which 251 cases were fetal kidney related abnormalities. Including ultrasound soft indicators, hydronephrosis and renal structural abnormalities. Ultrasound showed that there were 25 cases of fetal hyperechoic kidney, including 14 cases of IHK (Table 1) and 11 cases of NIHK (Table 2). All patients underwent at least two genetic tests and were followed up by telephone within 6 months to 1 year after the expected date of delivery, including newborn survival and urinary status. We recorded the condition of patients, and statistically analyzed the results of patient detection and prognosis. SPSS27.0 statistical software, Chi-square test was used to compare the differences between groups. Due to the small sample size, Fisher exact probability method was used, and *p* < 0.05 was considered statistically significant.

**TABLE 1:** Etiological and prognostic follow-up of IHK in our cohort.

	NO.	Age (y)	GW	Ultrasound results	Karyotype	CNV-seq	WES(Trio)	Outcomes
1	190034	26	25	Bilateral kidney echogenic, oligohydramnios	—	(−)	—	(−)
2	200244	31	28	Bilateral kidney echogenic	—	**1.del(17) (q12)** chr17:g34800000_36260000del,1.46 Mb,**p**; **2.dup(8) (q11.1)** chr8:g.46880000_47500000dup,0.62 Mb, **lb**	—	TOP
3	210040	38	19	Bilateral kidney echogenic	(−)	(−)	**1.*PKHD1*** chr6:51921499 NM_138694.3:c.1690C>T, (p.Arg564*), EX18/CDS17, (**het,p**)PKD type 4 with or without polycystic liver disease (OMIM:263200)/AR, Father het	TOP
							**2.*PKHD1*** chr6:51918033 NM_138694.3:c.1981A>C, (p.Thr661Pro), EX21/CDS20, (**het, vous**)AR, Mother het	
							**3.*NUP85*** chr17:73231283	
							NM_024844.3:c.1856C>T, (p.Thr619Ile), EX18/CDS18, (het, vous)Nephrotic syndrome type17, (OMIM:618176)/AR, Father het	
							**4.*NUP85*** chr17:73228963 NM_024844.3:c.1414A>G, (p.Ile472Val), EX15/CDS15, (het, vous) AR, Mother het	
4	210086	33	24	Bilateral kidney echogenic, oligohydramnios, consider infantile polycystic kidney disease	(−)	(−)	**1.*PKHD1*** chr6:51513947 NM_138694.3:c.11246C>T, (p.Pro3749Leu) EX62/CDS61 (het,vous) PKD type 4 with or without polycystic liver disease (OMIM:2632 00)/AR, Mother het	TOP
							**2.*PKHD1*** chr6:5152425 NM_138694.3:c.10679C>G, (p.Ser3560Cy s), EX61/CDS60 (het,vous)/AR, Father (het)	
5	210323	26	29	Bilateral kidney echogenic	—	(−)	**1.*PKD1*** chr16:2158269 NM_001009944.2:c.6899delG, (p.Cys2300Leufs*14) EX15/CDS15, (**het**/Father het/Mother wt,**p**) PKD type1, (OMIM:173900)/AD; **2.*PKD1*** chr16:2162862 NM_001009944.2:c.3088G>A, (p.Val1030Met) EX13/CDS13, (het/Father wt/Mother het vous) AD; **3.*COL4A3*** chr2:228131184–228131186 NM_000091.4:c.1367_1369del, ATC (p.Tyr456del)EX22/CDS22, (**het**/Father wt/Mother het, **lp**) Autosomal dominant Alport syndrome (OMIM:104200)/AD, Benign familial hematuria (OMIM:141200)/AD, Autosomal recessive Alport syndrome type 2 (OMIM:203780)/AR	ADPKD
6	220253	34	22	Bilateral kidney echogenic	(−)	dup (X) (p22.31p22.31)*1 1.77 Mb, **p**, X-linked ichthyosis	**1.*PKHD1*** chr6:51798938 NM_138694.3:c.6091del G, (p.Ala2031Leufs *2) EX37/CDS36, (het/Father het/Mother wt, p)PKD type 4 with or without more Cystic liver (OMIM:263200)/AR; **2.*PKHD1*** chr6:51927371 NM_138694.3:c.1064T>G (p.Val355Gly) EX14/CDS13, (het/Father wt/Mother het,vous) **supplement 1: *STS*** chrX:6968338–7268302 NM_000351.4: EX1-EX10E Del, (het/Father hem/Mother wt,p),X-linked ichthyosis (OMIM:308100)/XL	TOP
7	220302	30	18	Bilateral kidney echogenic	(−)	(−)	—	(−)
8	Y18002	30	20	Bilateral kidney echogenic	(−)	**del(4q13.3)**,130.91 kb, vous;**del(15q13.2)**, 200.68 kb, vous; **dup(15q13.3)** 489.55 kb, vous	—	(−)
9	Y19113	30	22	Bilateral kidney echogenic	T21	T21	—	TOP
10	Y19158	28	24	Bilateral kidney echogenic	(−)	(−)	—	(−)
11	Y19255	40	24	Bilateral kidney echogenic	T21	T21	—	TOP
12	Y20138	36	25	Bilateral kidney echogenic, Bilateral kidneys are full in shape	(−)	**dup(7p21.3)**,102.32 kb, vous **dup(17p12)**, 110.08 kb, vous	—	(−)
13	Y21263	34	27	Bilateral kidney echogenic	(−)	(−)	—	(−)
14	Y21447	31	19	Bilateral kidney echogenic, enlarged	(−)	**del(8p22p22)**, 205.37 kb vous	—	(−)

(−), nothing abnormal detected; TOP, termination of pregnancy; /, undetected; het, heterozygous; wt, wild type; hem, hemizygous; p, pathogenicity; lp, likely pathogenicity; b, benign; lb, likely benign; GW, gestational weeks; T, Trisomy; vous, variants of uncertain clinical significance.

**TABLE 2:** Etiological and prognostic follow-up of NIHK in our cohort.

N	NO.	Age (y)	GW	Ultrasound results	Karyotype	CNV-seq	WES (Trio)	Outcomes
1	170015	21	24	Fetal meningocele, hyperechoic bilateral kidneys, hexadactyly, Meckel-Gruber syndrome considered	—	umbilical cord **dup(1) (q21.1)** (145380001–145840000) 0.46 Mb, vous	umbilical cord: **1. *CC2D2A*** (het) chr4: 15565018 AR Meckel -Gruber Syndrome, **lp**; **2. *NUTM2B*/*IL17RD***, **lp**; **3. *DBT*/*NOS3***, **lp**	TOP
2	Q170004	28	21	High value of left ventricle, bilateral renal pelvis separation, bilateral renal parenchyma echo enhancement, excessive amniotic fluid	failed	(−)	—	TOP
3	180151	25	22	NT 5.6 mm, Cerebellar vermiform dysplasia, left cleft lip and palate, single atrium, endocardial pad defect, right ventricular double outlet, persistent left upper cavity, pulmonary artery stenosis, six fingers, toes, partial syndactyly possible, small omphalocele, umbilical cord cyst? The echo of both kidneys was enhanced	T13	T13	—	TOP
4	Q210001	22	25	Brain abnormalities: Dandy-Walker syndrome, arachnoid cysts; Cervical hygroma; Both kidneys increased echogenicity; Oligohydramnios	(−)	(−)	—	TOP
5	Y18243	34	22	The fetal right kidney is echogenic with multiple anechoic masses (considering the possibility of polycystic dysplasia); the left renal pelvis is separated, and the fetal heart rate is slightly faster	(−)	**dup(16p11.2p11.2)** 621.32 kb, vous	—	(−)
6	Y18280	27	22	Left kidney pelvic ectopic, slightly higher echo, mild hydrops; Fetal gallbladder morphology is slightly full	(−)	**del(14q31.3q31.1)** 303.82 kb, vous	—	(−)
7	Y18300	29	23	Fetal right lateral ventricle high value; Bilateral renal structural abnormalities: left renal corticomedul-medullary boundary blurred, parenchymal echo obviously enhanced; Multiple cysts (not excepting left renal polycystic dysplasia), renal pelvis separation; The cortical echo of the right kidney was increased, and the renal pelvis was separated	(−)	(−)	—	(−)
8	Y19125	31	19	NF thickening, neck and facial subcutaneous soft tissue thickening; Left nasal bone is short, right nasal bone is not explored (consider right nasal bone missing); Strong spot of left and right ventricle and mild regurgitation of tricuspid valve; The kidneys were highly echogenic and the liver was coarsely echogenic	T21	T21	—	TOP
9	Y19182	26	18	Blake cyst may be between the fourth ventricle and posterior fossa cisterna, and fetal septum pellucidum is small. Kidneys slightly larger; Fetal left ventricular hyperechoic spot	(−)	**del(9p23p23)** 338.97 kb, **lb**	—	TOP
10	Y21182	27	24	Uneven fetal development; Narrow septum pellucidum; Strong spot in right ventricle; Both kidneys are echogenic	(−)	**del(16p11.2p11.2)** 358.22 kb,**p**; **dup(5p15.2p15.2)** 178.09 kb, vous	—	TOP
11	Y21275	25	24	High posterior Angle of left ventricle, enhanced echo in both kidneys, separation of renal pelvis, small anechoic mass in right kidney, cyst considered	(−)	**del(17q12q12)** 1.83 Mb, **p**	—	TOP

(−), nothing abnormal detected; TOP, termination of pregnancy; /, undetected; het, heterozygous; wt, wild type; hem, hemizygous; p, pathogenicity; lp, likely pathogenicity; b, benign; lb, likely benign; GW, gestational weeks; T, Trisomy; vous, variants of uncertain clinical significance.

The bold value indicates the presence of microdeletions/microrepeats on the chromosome, or it may not be bold, just to be more eye-catching.

### Meta-analysis

#### Literature search

In February 2023, were searched by two researchers using combinations of the following keywords: “fetus,” “antenatal,” “prenatal,” fetus*,” “fetal,” “kidney,” “kidney*,” “hyperechoic” and “hyperechoic*.” For all data-bases, the last search was run on 28 February 2023.

The Chinese language databases Wanfang Data, China National Knowledge Infrastructure (CNKI), and China Biomedical Literature Database (CBM) (from 1 January 1990 to 28 February 2023) were searched by two researchers using the Chinese terms for “fetal,” “kidneys,” and “hyperechoic.” Additionally, English language databases PubMed, Embase, Cochrane Library and Web of Science (from 1 January 1945 to 28 February 2023) were searched by two researchers using combinations of the following keywords: “fetus,” “antenatal,” “prenatal,” fetus*,” “fetal,” “kidney,” “kidney*,” “hyperechoic” and “hyperechoic*.” The amount of amniotic fluid was assessed by a semiquantitative method. Diagnostic criteria were based on ultrasonographic assessment of AFV (amniotic fluid volume) or AFI (amniotic fluid index): AFV ≥ 8 cm or AFI ≥ 25 cm for polyhydramnios, and AFV ≤ 2 cm or AFI ≤ 5 cm for oligohydramnios (Phelan et al., 1987; Moore and Cayle, 1990). Kidneys were considered as hyperechogenic when the renal parenchyma was of greater echogenicity than adjacent liver tissue. The size of the kidneys was selected as standard deviations above or below the mean derived from the growth charts of Cohen et al. (1991) Renal enlargement was defined as kidney size ≥ 2 SD or a renal volume above the 90th percentile for gestational age (Chitty and Altman, 2003), kidney reduction was defined as kidney size ≤ 2 SD or a renal volume below the 10th percentile for gestational age, and between the two cutoff values was defined as normal kidney. Isolated hyperechogenic kidneys was defined by the absence of structural abnormalities. Review Manager 5.4.1 software was used for the meta-analysis to determine the incidence of chromosomal abnormalities and gene abnormalities in hyperechogenic kidneys, and the OR of the different outcomes and 95% confidence interval (CI) were calculated. Follow PRISMA (http://www.prisma-statement.org/) and MOOSE (Stroup et al., 2000) guidelines. We manually searched the reference lists of relevant articles and reviews for additional reports. The study has been registered in the PROSPERO database (registration No. CRD42023424469).

#### Inclusion criteria

Inclusion criteria were studies reporting fetal, neonatal, and infant outcomes. Renal hyperechogenicity was diagnosed on prenatal ultrasound. The primary outcomes were termination of pregnancy (TOP), intrauterine death (IUD) and neonatal death (NND).

#### Study selection

Screening was performed independently by two investigators based on inclusion and exclusion criteria. Any disagreements were resolved by discussion with a third researcher. The inclusion criteria is that patients with fetal renal hyperechogenicity confirmed by fetal ultrasound in the second or third trimester, including singleton and twin pregnancies.

Exclusion criteria were as follows: 1) Reviews and case reports; 2) animal research; 3) There was no further testing or follow-up outcome.

### Data extraction

Two researchers independently screened the literature, extracted and cross-checked the data. Differences, if any, are resolved through discussion or negotiation with a third party. During literature screening, the title of the paper was read first, and after excluding obviously irrelevant literature, the abstract and full text were further read to determine inclusion. Contact original study authors when necessary for information not identified but important to this study.

Data extraction included: 1) Basic information of the included studies: Research title, first author, published journals, etc. 2) the baseline characteristics of the research object and further detection methods; 3) Key elements of risk of bias assessment; 4) Outcome indicators of interest and follow-up prognosis data.

### Quality assessment of the selected articles

The Newcastle-Ottawa scale was used to evaluate the quality of the included literature, according to NOS, each study is judged on three broad perspectives: selection, comparability and results, i.e., the selection of the study groups; the comparability of the groups and the ascertainment of outcome of interest. Assessment of the selection of a study includes the evaluation of the representativeness of the exposed cohort, selection of the non-exposed cohort, ascertainment of exposure and the demonstration that the outcome of interest was not present at the start of study. Assessment of the comparability of the study includes the evaluation of the comparability of cohorts based on the design or analysis. Finally, the ascertainment of the outcome of interest includes the evaluation of the type of the assessment of the outcome of interest, its length and the adequacy of follow up. According to NOS, a study can be awarded a maximum of one star for each numbered item within the Selection and Outcome categories. A maximum of two stars can be given for Comparability (Wells et al., 2011). There are 8 items with a total score of 9 stars, studies with at least 6 stars rated as high quality.

### Statistical analysis

Review manager 5.4.1 software was used for statistical analysis. The single group rate was used as the effect analysis statistic, and its 95% confidence interval (CI) was given. The I2 was used to quantitatively determine the size of heterogeneity. The fixed effect and random effect models were used for meta-analysis. When *p* > 0.10 and I2< 50%, there was no statistical heterogeneity among the results of each study, then the fixed effect model was used for meta-analysis. Otherwise, there was statistical heterogeneity among the results of each study, and the sources of heterogeneity were further analyzed. After excluding the influence of obvious clinical heterogeneity, a random effect model was used for meta-analysis. The test level for the meta-analysis was set at *α* = 0.05. Methods such as subgroup analysis or sensitivity analysis were used to deal with significant clinical heterogeneity, or only descriptive analyses were performed.

## Results

### Cohort study

**FIGURE 1:**
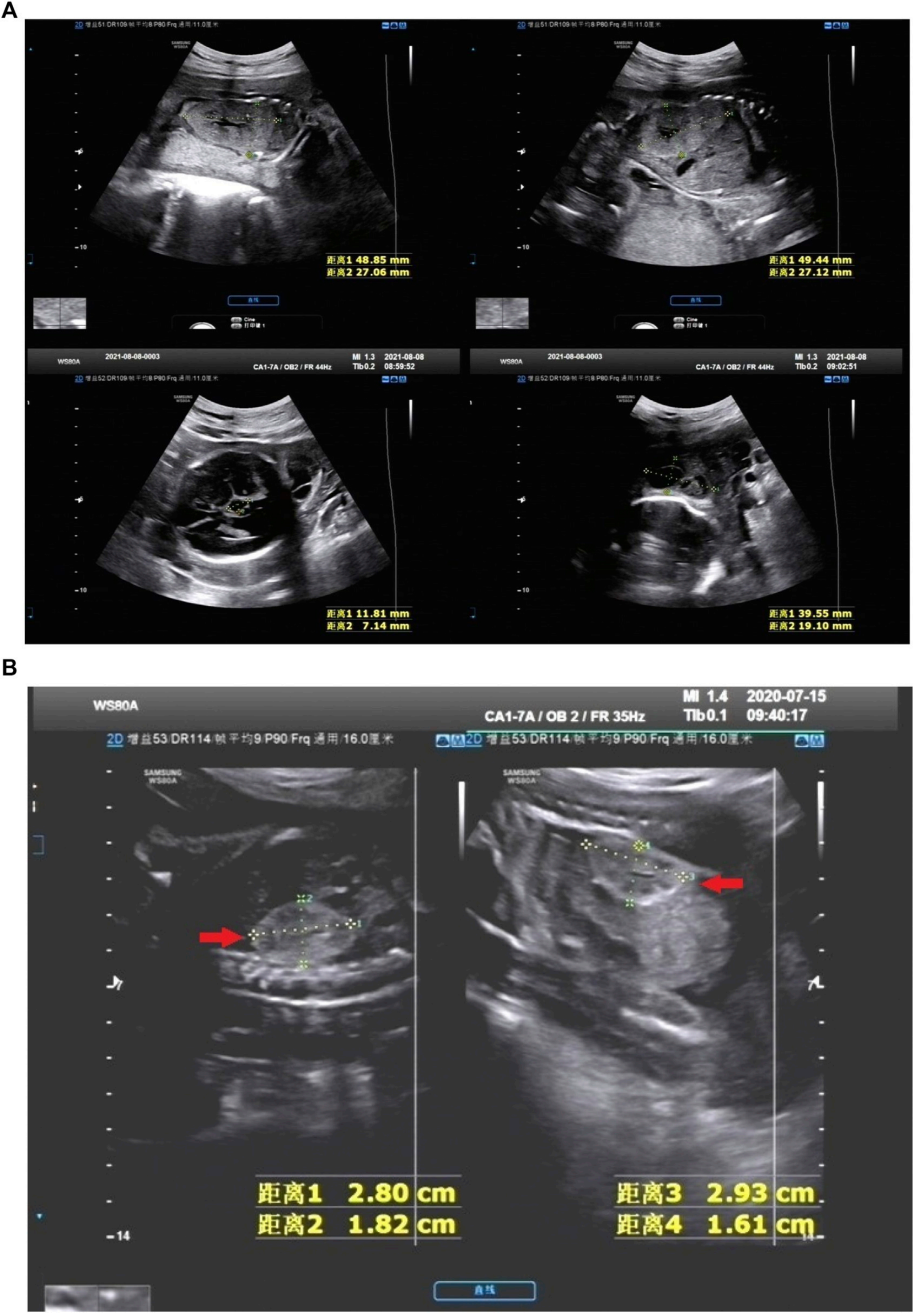
**(A)** NO. Q210001 in NIHK group, the ultrasound images of 28 weeks showed the size of the left kidney was 48.85 mm*27.06 mm and that of the right kidney was 49.44 mm*27.12 mm, the echo of the renal parenchyma was thickened and enhanced; An 11.81 mm*7.14 mm anechoic area was found in the midline below the third ventricle, Dandy-Walker syndrome with arachnoid cyst was considered; There was a 39.55 mm*19.10 mm anechoic area in the left posterior part of the neck, which was separated by a light band, It was considered as a cystic hygroma of the neck. This case was accompanied by oligohydramnios. **(B)** NO. 200244 in IHK group, the ultrasound images of 25 weeks showed bilateral hyperechoic kidneys, the size of the right kidney was 28 mm*18 mm and that of the left kidney was 29 mm*16 mm, within normal limits.

There were 25 HEK cases in our cohort, including 14 IHK cases and 11 NIHK cases. Among all the cases, 4 cases were unable to perform amniotic fluid karyotype analysis due to gestational age over 25 weeks. Karyotyping failed in 1 case (NO. Q170004), the fetus was terminated because of multiple malformations, as shown in Figure 1A for the ultrasound images. 4 cases (20%) of aneuploidy were detected in 20 cases, of which 18% (2/11) were IHK and 22% (2/9) were NIHK. Copy number variation sequencing (CNV-seq) based on next-generation sequencing (NGS) was used to detect copy number variations (CNVs) in all 25 HEK cases, and 8 cases were pathogenic and suspected pathogenic, among which the detection rate of IHK was 29% (4/14), including 1 fetus (NO. 200244) of 17q12 deletion syndrome, the ultrasound image is shown in Figure 1B, which shows enhanced echo in both kidneys. A pathogenic CNV associated with X-linked ichthyosis was also found in case 6 (NO. 220253). The results of the other 2 cases (NO. Y19113 and NO. Y19255) were consistent with the karyotype test, and both were trisomy 21 syndrome. The abnormal detection rate of CNV in NIHK group was 37% (4/11), including 1case (NO. Y21275) of 17q12 deletion syndrome. The overall detection rate of variants of unknown clinical significance was 24%, only 1 case of benign or suspected benign variants was detected, and the detection rate was 4% in the NIHK group. There were 10 cases with no definite abnormality, accounting for 40%. A total of 5 cases underwent whole exome sequencing (WES), and no kidney-related pathogenic variants were detected in their karyotypes and CNV-seq. The results of WES in 4 cases of IHK group showed that 3 cases had compound heterozygosity of *PKHD1* gene. The case No. 5 of IHK has a compound heterozygous *PKD1* genotype, involving a pathogenic and an uncertain significance variant, while the fetus is also heterozygous for a *COL4A3* gene variant responsible of the autosomal dominant form of Alport syndrome. Only 1 case of WES in NIHK group, the results showed that the suspected pathogenic gene *CC2D2A* was detected, which was related to Meckel-Gruber Syndrome. The follow-up results showed that 14 cases (56%) had termination of pregnancy (TOP), the TOP rate was 43% (6/14) in the IHK group and 73% in the NIHK group. 11 patients survived well, including 1 case with ADPKD, and no neonatal deaths occurred. In the IHK group, Case 1, and Case 4 had less amniotic fluid, Case 4 was diagnosed with *PKHD1* and the pregnancy was terminated, and case 1 was born normally. Case 14 had slightly enlarged kidneys and was born normally. In the NIHK group, the pregnancy was terminated in case 4 with oligohydramnios and case 9 with renal enlargement. However, there was no significant difference in the detection rate of abnormal karyotypes and CNV-seq between IHK and NIHK groups (Table 3).

**TABLE 3:** Etiology and outcome statistics of IHK and NIHK in our cohort.

Testing and follow-up		IHK (14)		NIHK (11)		Total (25)		χ^2^	p
		Number	p1	Number	p2	Number	p3		
karyotype	abnormal	2	0.18 (2/11)	2	0.22 (2/9)	4	0.2	0.318	>0.05
	normal	9	0.82 (9/11)	7	0.78 (7/9)	16	0.8		
	undetected	3	—	2 (1failed)	—	5	—		
CNV-seq	p/lp	4	0.29	4	0.37	8	0.32	3.53	>0.05
	vous	3	0.21	3	0.27	6	0.24		
	b/lb	0	0	1	0.09	1	0.04		
	normal	7	0.50	3	0.27	10	0.40		
WES	abnormal	4	1	1	1	5	1	—	—
	undetected	10	—	10	—	20	—		
follow-up	TOP	6	0.43	8	0.73	14	0.56	—	>0.05
	Survived well	8 (1ADPKD)	0.57	3	0.27	11	0.44		

p, pathogenicity; lp, likely pathogenicity; b, benign; lb, likely benign; TOP, termination of pregnancy; ADPKD, autosomal dominant polycystic kidney disease.

### Results of literature screening and characteristics of included studies

**FIGURE 2:**
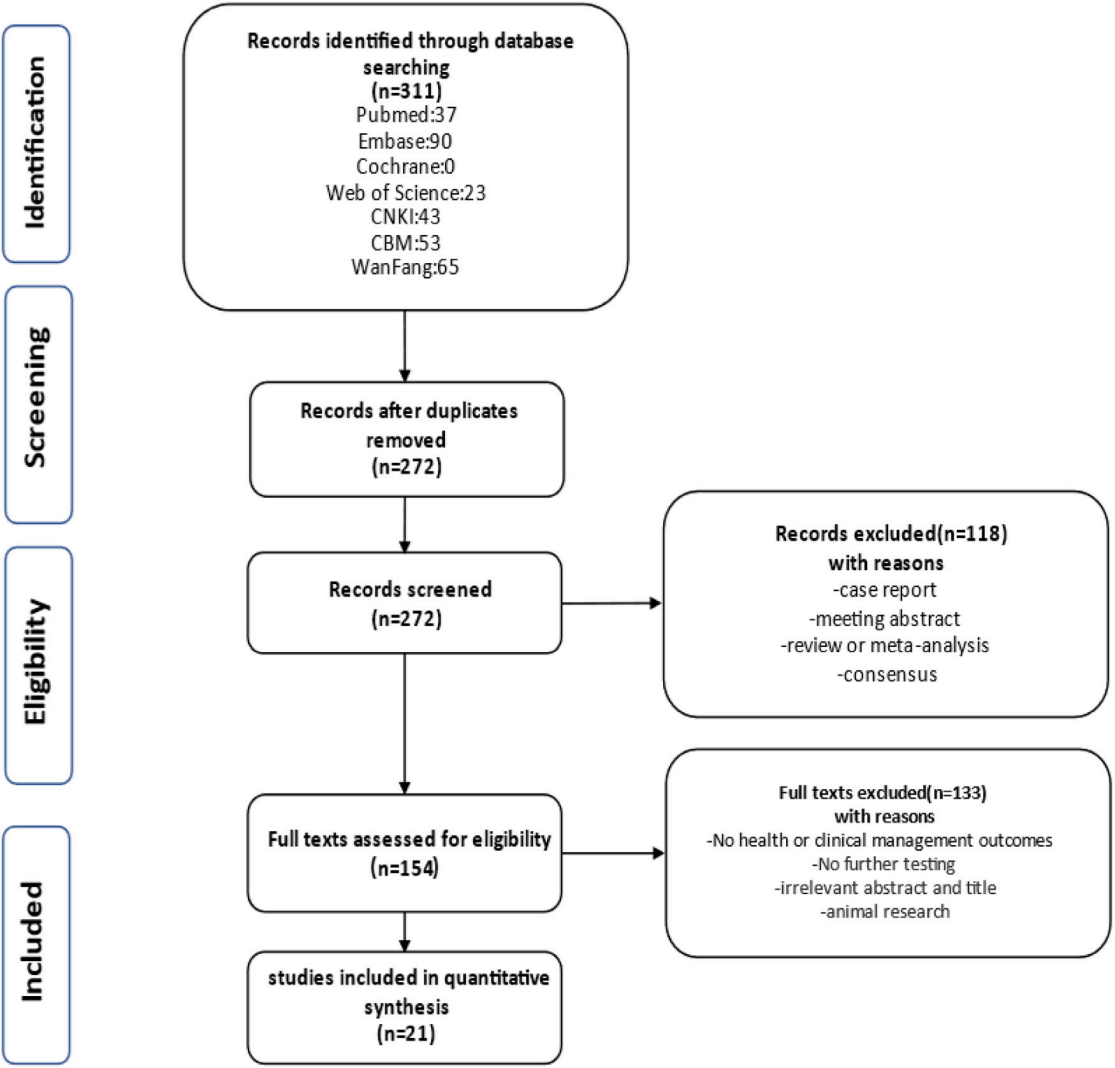
Flowchart of study selection.

Retrieving articles according to predefined search terms, among the 311 Chinese and English articles identified, we eliminated 39 duplicate articles, 118 reviews, consensus and case reports, and 115 articles with irrelevant abstract and title, and finally selected the remaining 39 articles. After further research, a total of 21 articles (Decramer et al., 2007; Su et al., 2022; Li et al., 2007; Morr et al., 2022, Chaumoitre et al., 2006; Digby et al., 2021; Mashiach et al., 2005; Estroff et al., 1991; Yulia et al., 2021; Shuster et al., 2019; Deng et al., 2022; Gilboa et al., 2016; Tsatsaris et al., 2002; Emmanuelli et al., 2010; Carr et al., 1995; Heidet et al., 2010; Li et al., 2020; Zhang et al., 2017; Huang, 2014; Xie et al., 2022; Chen et al., 2022) were included in the final analysis (Figure 2; Table 4). Overall 1,178 cases arising from these studies were considered in the present meta-analysis. Table 4 records the basic information of the included studies, including author, year, nationality, study design, gestational age at prenatal ultrasound diagnosis, outcome observation indicators, and follow-up time limit. The NOS was used to the quality assessment of the included studies (Table 5). The studies showed good overall scores for the selection and comparability of study groups, and for the identification of outcomes of interest; Studies were of high overall quality with a minimum score of 6 stars. Most of these studies had retrospective designs and some had small sample sizes, which may have contributed to the heterogeneity of the observed results. The total number of IHK and NIHK fetuses and the number of abnormal karyotypes, CMA and monogenic etiologies detected in each group are shown in Table 6.

**TABLE 4:** List of the included studies.

Author	Year	Country	Study design	Study period	GW	Outcomes observed	Duration of follow-up	n
Yulia, A	2021	United Kingdom	Retrospective	2002–2017	21 (13–37)	US, creatinine, hypertension requiring medication or major kidney surgery, dialysis or kidney transplant	up to 1 year of age	316
DigbyADDIN	2021	Canada	Retrospective	2013–2019	21 (12–39)	US, blood pressure, creatinine/eGFR or urinalysis, kidney surgery, dialysis or kidney transplant	2–71 months	31
Emmanuelli, V	2010	France	Retrospective	1997–2008	25 (21–37)	US, Blood pressure and renal function	12–132 months	17
Decramer, S	2007	France	Retrospective	1987–2005	26.0 ± 5.68 (18–35)	US, serum creatinine, AST, ALT, GGT, GFR, pancreatic enzymes, blood glucose, insulin, and glycosylated hemoglobin	69.16 (6–180) months	62
Tsatsaris, V	2002	France	Prospective	1985–1996	18–37	US, postnatal US, blood pressure, serum creatinine, proteinuria, renal biopsy when available	84 (34–132) months	43
Estroff, J. A	1991	United States	Retrospective	1987–1990	16–40	postnatal US, serum creatinine	—	19
Su, J	2022	China	Retrospective	2013–2019	14–36	US	—	48
Shuster, S	2019	Canada	Retrospective	2000–2015	17	US	—	52
Heidet, L	2010	France	Retrospective	before 2019	12–32	US, serum creatinine	1–17 years	55
Carr, M. C	1995	United States	Retrospective	1990–1993	—	US, serum creatinine, electrolytes, A voiding cystourethrogram or radionuclide cystogram	3 years	8
Mashiach, R	2005	Israel	Retrospective	1996–2002	16–40	US, serum creatinine, electrolytes	3 years	7
Deng, L	2022	China	Retrospective	2016–2020	28–42	postnatal neonatal renal ultrasound examination, blood pressure, renal function, urine routine, growth and development	1 month-3 years 7 months	28
Gilboa, Y	2016	Israel	Prospective	2006–2015	22–33	US, renal function	4 years	7
Morr, A	2022	Germany	Retrospective	2000–2018	26 (12–34)	US, renal function	2–16 years	23
K. CHAUMOITRE	2006	France	Retrospective	1990–2002	13–36	US	0–7 years	30
Dongqing Xie	2022	China	Retrospective	2015–2018	17–38	US	11 month-3 years 10 months	26
Fei Chen	2022	China	Retrospective	2014–2020	27 (13–33)	US	—	74
Chunling Li	2020	China	Retrospective	2015–2019	15–37	US	—	210
Junhua Huang	2014	China	Retrospective	2011–2013	27–39	US, fetal autopsy	—	26
Xiaoxiao Zhang	2017	China	Retrospective	2009–2015	20–38	US	0–6 years	65
Hui Li	2007	China	Retrospective	2000–2004	25–39	prenatal US features, the neonatal renal ultrasound, blood pressure, renal function, urine routine, growth and development status, renal biopsy if necessary	2–6 years	31

US, ultrasound. GW, gestational weeks.

**TABLE 5:** Quality assessment of the included studies according to NOS.

Study	Selection	Comparability	Outcome
Yulia et al. (2021)	★★★	★	★★★
Digby et al. (2021)	★★★	★★	★★★
Emmanuelli et al. (2010)	★★	★★	★★★
Decramer et al. (2007)	★★★	★★	★★
Tsatsaris et al. (2002)	★★	★★	★★★
Estroff et al. (1991)	★★★	★★	★★
Su et al. (2022)	★★★	★	★★
Shuster et al. (2019)	★★	★★	★★
Heidet et al. (2010)	★★★	★	★★
Carr et al. (1995)	★★	★	★★★
Mashiach et al. (2005)	★★★	★★	★★★
Deng et al. (2022)	★★★	★★	★★★
Gilboa et al. (2016)	★★★	★	★★★
Morr et al. (2022)	★★	★★	★★★
Chaumoitre et al. (2006)	★★	★★	★★★
Xie et al. (2022)	★★★	★★	★★★
Chen et al. (2022)	★★★	★	★★
Li et al. (2020)	★★★	★★	★★
Huang (2014)	★★	★	★★
Zhang et al. (2017)	★★★	★	★★★
Li et al. (2007)	★★★	★★	★★★

The Newcastle-Ottawa scale (NOS). Selection: 1. Representative for the population; 2. Ascertainment of exposure; 3. Consecutive patients; 4. Outcome not present at the start of the study. Outcomes:1. Assessment of outcome well performed; 2. select an adequate follow up period; 3. Adequacy of follow up.

**TABLE 6:** The detection of chromosomal and monogenic etiologies in IHK and NIHK.

Study	Total sample	N of IHK	N of NIHK	N of karyotypes abnormalities		N of CMA abnormalities		N of monogenic etiologies	
				IHK	NIHK	IHK	NIHK	IHK	NIHK
Yulia et al., 2021	316	—	—	—	—	—	—	—	—
Digby et al. (2021)	31	19	11	0/2	3/6	1/2	6/11	1/1	1/1
Emmanuelli et al. (2010)	17	17	—	0/9	—	—	—	—	—
Decramer et al. (2007)	62	62	—	—	—	—	—	16/62	—
Tsatsaris et al. (2002)	43	43	—	—	—	—	—	—	—
Estroff et al. (1991)	19	19	—	—	—	—	—	—	—
Su et al. (2022)	48	36	12	—	—	16/36	3/12	—	—
Shuster et al. (2019)	52	52	—	0/18	—	1/34	—	22/22	—
Heidet et al. (2010)	55	55	—	—	—	—	—	34/55	—
Carr et al. (1995)	8	8	—	—	—	—	—	—	—
Mashiachet al. (2005)	7	7	—	0/3	—	—	—	—	—
Deng et al. (2022)	28	8	20	0/8	3/20	2/8	5/19	3/6	10/10
Gilboa et al. (2016)	7	7	—	—	—	5/7	—	—	—
Morr et al. (2022)	23	—	23	—	2/14	—	—	—	6/6
Chaumoitre et al. (2006)	30	—	30	—	—	—	—	—	—
Xie et al. (2022)	26	15	11	—	1/5	—	—	—	11/11
Chen et al. (2022)	74	—	74	—	—	—	5/17	—	—
Li et al. (2020)	210	86	124	—	—	9/32	—	—	—
Huang (2014)	26	—	26	—	4/26	0/1	—	—	—
Zhang et al. (2017)	65	31	34	0/6	0/14	—	0/2	1/1	—
Li et al. (2007)	31	25	6	—	3/6	—	—	—	—
total	1,178	490	371	0/46	16/91	34/120	19/61	77/147	28/28

### Positive rate of karyotype in NIHK

**FIGURE 3:**
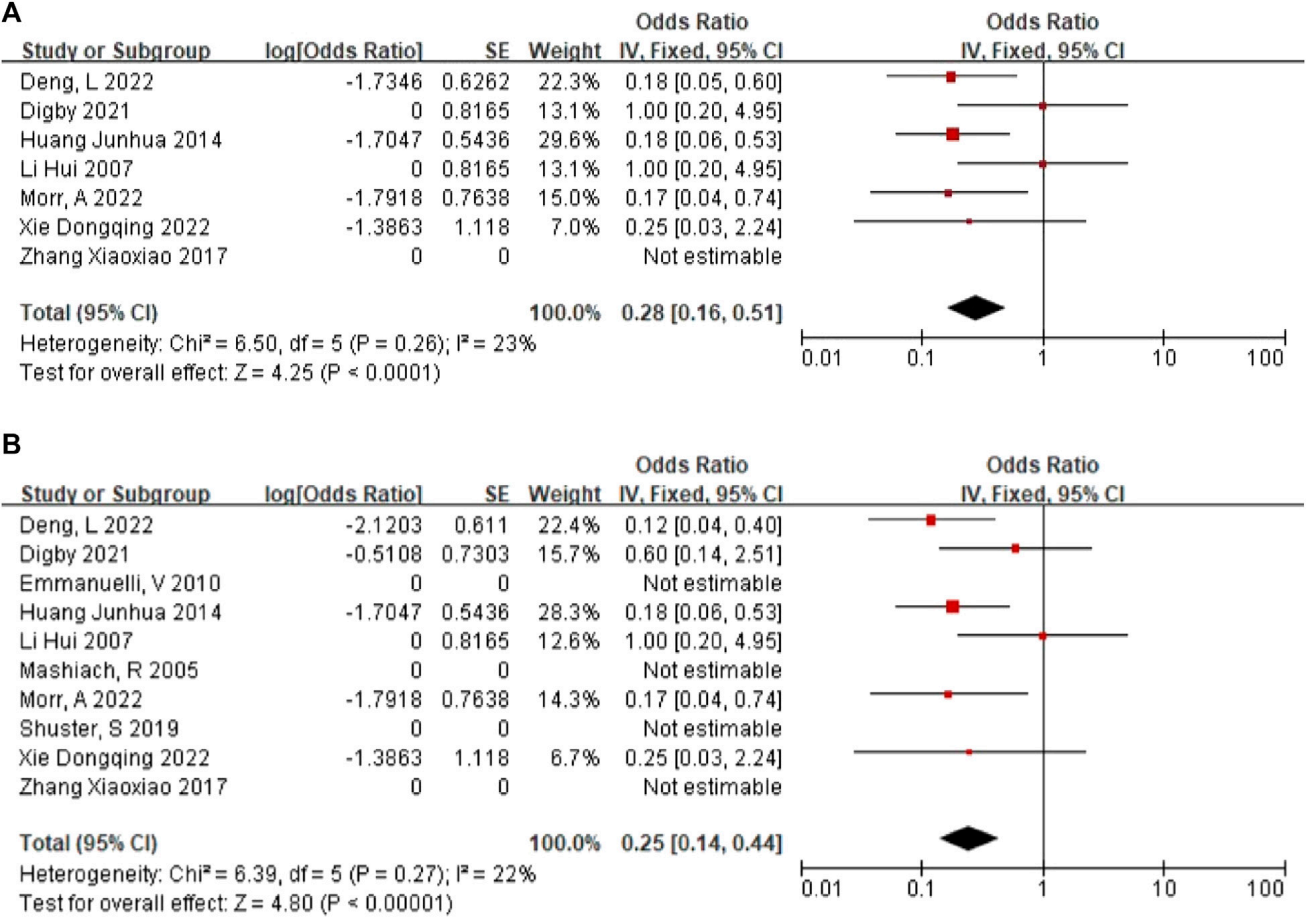
**(A)** The positive rate of karyotype in NIHK. **(B)** The positive rate of karyotype in HEK.

In 6 articles (Li et al., 2007; Huang, 2014; Digby et al., 2021; Deng et al., 2022; Morr et al., 2022; Xie et al., 2022), a total of 91 NIHK fetus were included, of which 16 abnormal karyotypes were detected. The heterogeneity test for all studies yielded I2 = 23%. We choose the results of the fixed effects model. Meta-analysis showed that positive rate of karyotype in NIHK with the OR of 0.28, (95% CI 0.16–0.51) (Figure 3A), the *p*-value was 0.22 (95% CI 0.14–0.34).

### Positive rate of karyotype in HEK

In 10 articles (Mashiach et al., 2005; Li et al., 2007; Emmanuelli et al., 2010; Huang, 2014; Zhang et al., 2017; Shuster et al., 2019; Digby et al., 2021; Deng et al., 2022; Morr et al., 2022; Xie et al., 2022), a total of 137 HEK fetus were included, of which 16 abnormal karyotypes were detected. The heterogeneity test for all studies yielded I2 = 22%. We choose the results of the fixed effects model. Meta-analysis showed that positive rate of karyotype in HEK was 32%, with the OR of 0.25, (95% CI 0.14–0.44) (Figure 3B), the *p*-value was 0.20 (95% CI 0.12–0.31).

### Positive rate of CMA in NIHK

**FIGURE 4:**
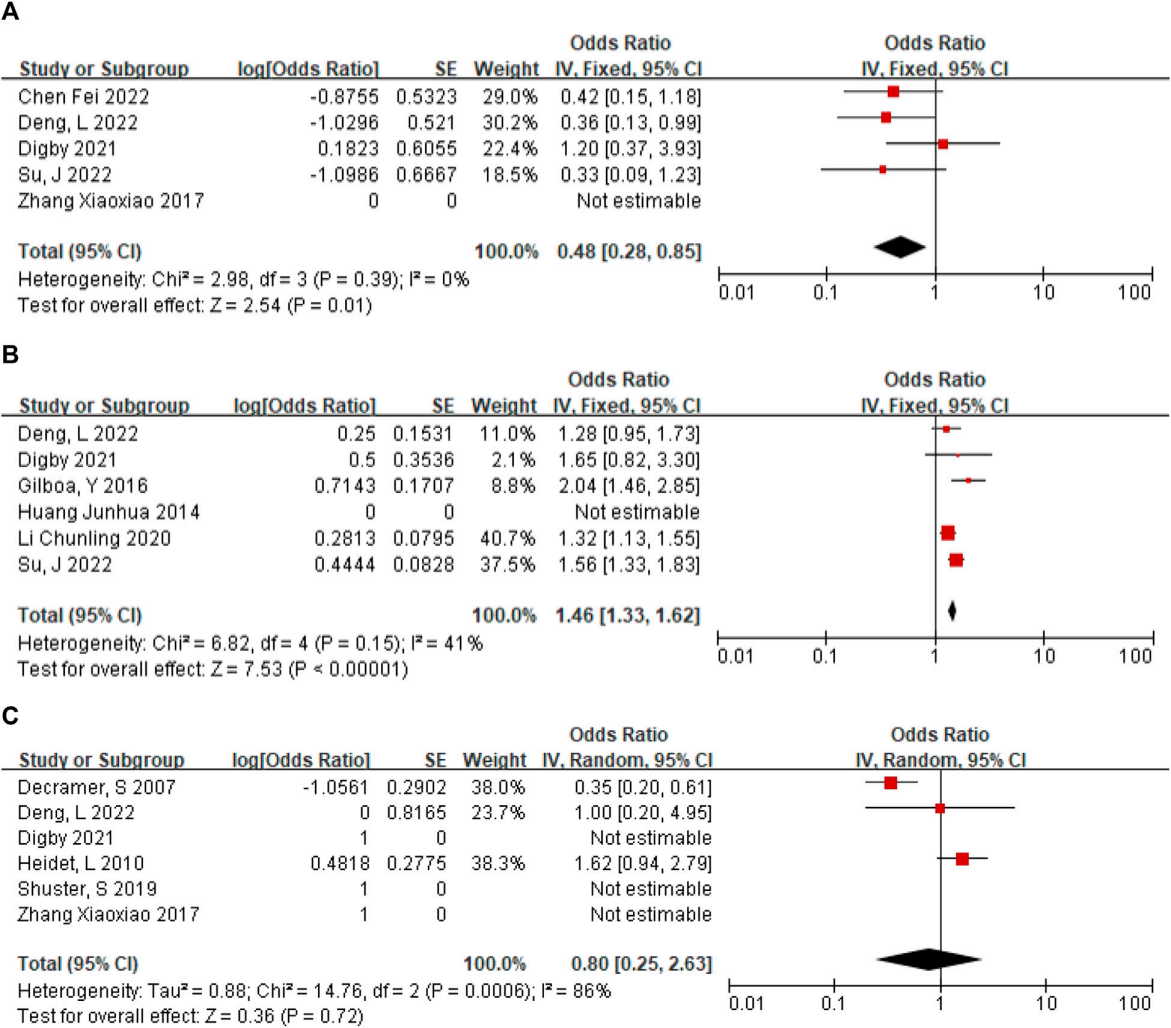
**(A)** The positive rate of CMA in NIHK. **(B)** The positive rate of CMA in IHK. **(C)** Positive rate of monogenic etiologies in IHK.

In 5 articles (Su et al., 2022; Digby et al., 2021; Deng et al., 2022; Zhang et al., 2017; Chen et al., 2022), a total of 61 NIHK fetus were included, of which 19 CMA abnormalities were detected. The heterogeneity test for all studies yielded I2 = 0%. We choose the results of the fixed effects model. Meta-analysis showed that the positive rate of CMA in NIHK patients with the OR of 0.48, (95% CI 0.28–0.85) (Figure 4A) the *p*-value was 0.32 (95% CI 0.22–0.46).

### Positive rate of CMA in IHK

A total of 120 patients with IHK were included in the 6 articles (Huang, 2014; Gilboa et al., 2016; Li et al., 2020; Digby et al., 2021; Deng et al., 2022; Su et al., 2022), among which 33 cases of CMA abnormalities were detected. The heterogeneity test for all studies was I2 = 41%. We choose the results of the fixed effects model. Meta-analysis showed that the positive rate of CMA in IHK patients with the OR of 1.46 (95% CI 1.33–1.62) (Figure 4B). The *p*-value was 0.59 (95% CI 0.57–0.62).

### Positive rate of monogenic etiologies in IHK

A total of 147 cases of HEK fetuses were included in the 6 articles (Decramer et al., 2007; Digby et al., 2021; Shuster et al., 2019; Deng et al., 2022; Heidet et al., 2010; Zhang et al., 2017), of which 77 cases were found to have monogenic etiologies by prenatal diagnosis or autopsy. The heterogeneity test was I2 = 86% for all studies. We choose the results of the random effects model. The positive rate of monogenic etiologies in HEK patients was 44%, with OR of 0.80 (95% CI 0.25–2.63) (Figure 4C).

### Subgroup analysis

Subgroup analysis of mortality by different amniotic fluid volume in IHK showed the total heterogeneity I^2^ = 74% and subgroup heterogeneity I^2^ = 48.4%, pooled OR 0.31 (95% CI 0.15–0.62); However, in NIHK, the total heterogeneity I2 = 68%, and the heterogeneity of subgroups I2 = 81.8%, pooled OR 0.54 (95% CI 0.34–0.88) (Supplementary Figures S1, S2). In subgroup analyses based on kidney size differential mortality showed the total heterogeneity and subgroup heterogeneity were high, as follows: IHK total I2 = 77%, subgroup I2 = 94.5%; NIHK total I2 = 87%, subgroup I2 = 76.3% (Supplementary Figures S3, S4). The total number of fetuses and deaths in each group, the OR, 95% CI, and *p* values are recorded in Table 7.

**TABLE 7:** Mortality analysis of different amniotic fluid volume and kidney size in IHK and NIHK.

	IHK mortality					NIHK mortality				
	Studies	Fetuses	OR	I^2^ (%)	p	Studies	Fetuses	OR	I^2^ (%)	p
Value of amniotic fluid										
AFV ≤ 2 cm or AFI ≤ 5 cm	5	52/150	0.60 [0.42, 0.85]	49	0.38	4	128/278	0.85 [0.67, 1.08]	34	0.46
Normal	4	10/109	0.15 [0.03, 0.79]	76	0.13	3	17/79	0.36 [0.21, 0.64]	41	0.26
AFV ≥ 8 cm or AFI ≥ 25 cm	0	0	0	—	0	1	3/23	0.15 [0.04, 0.50]	—	0.13
Kidney size										
Enlarged	6	57/157	0.56 [0.33, 0.98]	51	0.36	3	43/79	1.71 [1.56, 1.89]	91	0.63
Normal	4	7/119	0.07 [0.03, 0.15]	0	0.07	2	15/56	0.29 [0.02, 3.80]	90	0.22
Reduction	0	0	0	—	0	1	1/30	0.03 [0.00, 0.25]	—	0.03

### Pooled proportions of the adverse outcomes

The mortality of IHK and NIHK mainly includes termination of pregnancy (TOP), intrauterine death (IUD), neonatal death (ND), and even child death during long-term follow-up. 15 studies (Estroff et al., 1991; Tsatsaris et al., 2002; Mashiach et al., 2005; Decramer et al., 2007; Li et al., 2007; Emmanuelli et al., 2010; Heidet et al., 2010; Gilboa et al., 2016; Zhang et al., 2017; Shuster et al., 2019; Li et al., 2020; Digby et al., 2021; Yulia et al., 2021; Deng et al., 2022; Su et al., 2022) recorded the TOP 111 cases of 528 IHK fetuses, with the pooled OR of 0.26 (95% CI 0.17–0.40); Among 579 NIHK fetuses in 11 studies (Su et al., 2022; Morr et al., 2022, Chaumoitre et al., 2006; Digby et al., 2021; Yulia et al., 2021; Deng et al., 2022; Li et al., 2020; Zhang et al., 2017; Huang, 2014; Xie et al., 2022; Chen et al., 2022), 318 fetuses were terminated (TOP), with the pooled OR of 1.72 (95% CI 1.59–1.86); There was no intrauterine death in IHK fetus during pregnancy. Of 273 NIHK fetuses in the 2 studies (Yulia et al., 2021; Chen et al., 2022), 6 cases had intrauterine death, with the OR of 0.02 (95% CI 0.01–0.05), heterogeneity I2 = 0; There were 39 neonatal deaths in 328 IHK fetuses in 9 studies (Estroff et al., 1991; Tsatsaris et al., 2002; Mashiach et al., 2005; Decramer et al., 2007; Li et al., 2007; Emmanuelli et al., 2010; Zhang et al., 2017; Shuster et al., 2019; Yulia et al., 2021), with the OR of 0.15 (95% CI 0.10–0.24), I2 = 30%; There were 413 NIHK fetuses in 6 studies (Morr et al., 2022, CHAUMOITRE et al., 2006; Digby et al., 2021; Yulia et al., 2021; Li et al., 2020; Huang, 2014), 43 of them had neonatal death, with the OR of 0.12 (95% CI 0.06–0.26), I2 = 65%; 3 of 31 IHK fetuses in 1 study (Li et al., 2007) and 1 of 30 NIHK fetuses in 1 study (CHAUMOITRE et al., 2006) had childhood death, with the OR of 0.11 (95% CI 0.03–0.35) and 0.03 (95% CI 0.00–0.25), respectively. For the survival of *postpartum* neonates and children, such as the need for transplantation surgery, dialysis treatment, hypertension and diabetes drug treatment, there were 30 cases of 147 IHK fetuses in 6 studies (Estroff et al., 1991; Tsatsaris et al., 2002; Emmanuelli et al., 2010; Shuster et al., 2019; Yulia et al., 2021; Su et al., 2022), with the OR of 0.28 (95% CI 0.17–0.46), I2 = 20%. Among 93 NIHK fetuses in 6 articles (Su et al., 2022; Morr et al., 2022, CHAUMOITRE et al., 2006; Digby et al., 2021; Yulia et al., 2021; Deng et al., 2022), 20 cases had *postpartum* abnormalities, the OR 1.28 (95% CI 1.12–1.47), I2 = 30% (Table 8).

**TABLE 8:** Pooled proportions of the different adverse outcomes in fetuses.

	IHK					NIHK				
	Studies	Fetuses	Or (95% CI)	I^2^ (%)	p	Studies	Fetuses	Or (95% CI)	I^2^ (%)	p
Mortality										
TOP	15	111/528	0.26 [0.17, 0.40]	68	0.21	11	318/579	1.72 [1.59, 1.86]	69	0.63
IUD	0	0	0	—		2	6/273	0.02 [0.01, 0.05]	0	0.02
ND	9	39/328	0.15 [0.10, 0.24]	30	0.13	6	43/413	0.12 [0.06, 0.25]	65	0.11
Death in Childhood	1	3/31	0.11 [0.03, 0.35]	—	0.1	1	1/30	0.03 [0.00, 0.25]	—	0.03
Postnatal outcome										
Intervention	6	30/147	0.28 [0.17, 0.46]	20	0.22	6	20/93	0.40 [0.18, 0.92]	40	0.29

TOP, Termination of pregnancy; IUD, Intrauterine death; ND, neonatal death; Intervention, Need for surgery or medical treatment (hypertension, diabetes, dialysis or transplantation in renal failure, etc.).

### Pooled proportions for the etiology in IHK

Due to the uncertainty of intrarenal and extrarenal abnormalities, the diagnosis of NIHK is also diverse, involving various systems of the whole body, including various chromosomal abnormalities and even monogenic etiologies, while in IHK, the causes of diagnosis are relatively limited. Among 232 fetuses in 7 articles (Tsatsaris et al., 2002; Mashiach et al., 2005; Emmanuelli et al., 2010; Shuster et al., 2019; Li et al., 2020; Digby et al., 2021; Deng et al., 2022), 37 fetuses were diagnosed as ADPKD. There were 51/243 cases of ARPKD in 7 studies (Estroff et al., 1991; Tsatsaris et al., 2002; Mashiach et al., 2005; Emmanuelli et al., 2010; Shuster et al., 2019; Li et al., 2020; Digby et al., 2021). Three out of 93 cases of MCKD were reported in 2 studies (Mashiach et al., 2005; Li et al., 2020). The number of *HNF1B* variants diagnosed in 5 articles (Heidet et al., 2010; Gilboa et al., 2016; Li et al., 2020; Digby et al., 2021; Deng et al., 2022) was 47/175. One article (Li et al., 2020) reported that 4 aneuploidy cases were detected in 86 IHK fetuses. Two BBS were detected in 36 fetuses in the 2 articles (Emmanuelli et al., 2010; Digby et al., 2021). There were also 3 articles (Estroff et al., 1991; Tsatsaris et al., 2002; Deng et al., 2022) in which 27 other abnormalities were detected in 70 fetuses, It mainly includes *WTX* mutation, renal tubular disease, Familial nephroblastoma, AD CAKUT, Type II Ivemark Syndrome, renal agenesis, Beckwith-Wiedemann syndrome; In 7 studies (Estroff et al., 1991; Tsatsaris et al., 2002; Mashiach et al., 2005; Emmanuelli et al., 2010; Gilboa et al., 2016; Li et al., 2020; Deng et al., 2022), 42 out of 187 fetuses eventually returned to normal, hyperechogenicity disappeared; The OR (95% CI) and *p*-values are shown in Table 9, the diagnosis was made by ultrasound or pathology or gene diagnosis.

**TABLE 9:** Pooled proportions for the etiology in IHK.

Outcome	Studies	Proportion of deaths	Or (95% CI)	I^2^ (%)	p
ADPKD	7	37/232	0.21 [0.14, 0.32]	20	0.17
ARPKD	7	51/243	0.28 [0.12, 0.66]	78	0.22
MCDK	2	3/93	0.09 [0.03, 0.33]	86	0.08
*HNF1B* mutation	5	47/175	0.35 [0.06, 1.98]	91	0.26
Aneuploidy	1	4/86	0.05 [0.02, 0.13]	—	0.05
BBS	2	3/36	0.10 [0.03, 0.32]	0	0.09
Others^a^	3	27/70	0.43 [0.11, 1.65]	78	0.30
normal	7	42/187	0.30 [0.21, 0.43]	0	0.23

^a^ Others contained *WTX*, mutation, renal tubular disease, Familial nephroblastoma; AD, CAKUT, Type II, ivemark syndrome, renal agenesis, Beckwith-Wiedemann syndrome. BBS: Bardet-Biedl syndrome.

### Mortality for poor or absent corticomedultural differentiation (CMD)

**FIGURE 5:**
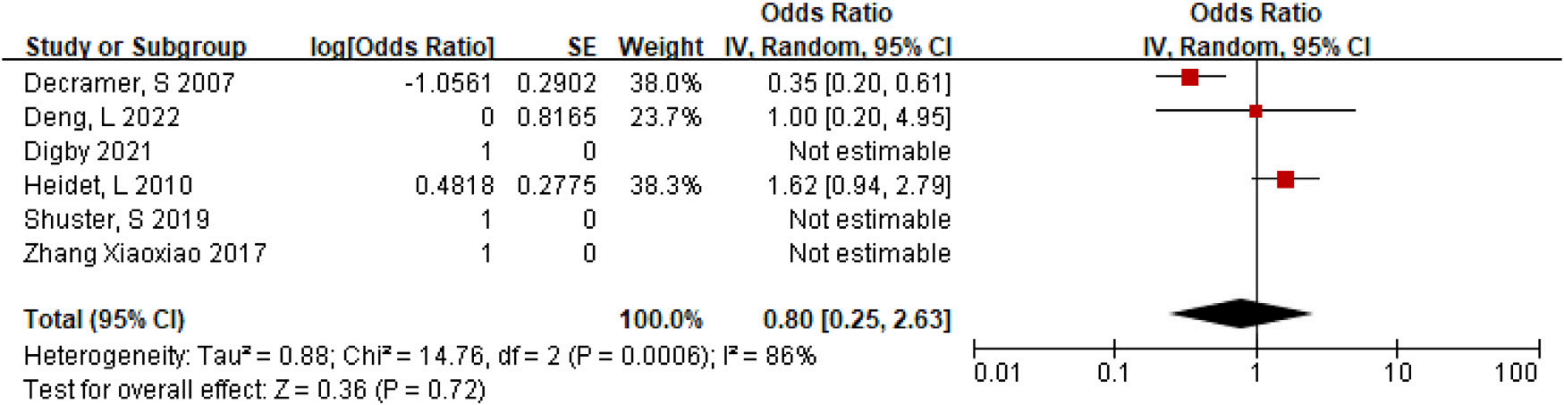
Mortality for poor or absent CMD.

Only a few studies (Decramer et al., 2007, CHAUMOITRE et al., 2006; Deng et al., 2022) reported renal CMD and the mortality of fetuses with poor or absent renal corticomedultural differentiation was calculated, with an OR of 1.87 (95% CI 1.66–2.11), heterogeneity I^2^ = 7% (Figure 5), Thus, the mortality rate of fetuses with poor or absent renal CMD is 65%.

### Sensitivity analysis

Sensitivity analyses were performed by individually excluding each included study to determine whether the combined results of the other studies were stable, and look for sources of heterogeneity. Sensitivity analysis was performed for data with >50% heterogeneity, as shown in Supplementary Figures S5–S7. The positive rate of monogenic etiologies in IHK with the OR of 0.45 (95% CI 0.19–1.08), *p*-value was 31%. In IHK fetuses, the mortality rate of oligohydramnios with an OR of 0.52 (95% CI 0.30–0.90), I2 = 49%, *p*-value was 34%; the mortality rate of normal amniotic fluid volume with an OR of 0.08 (95% CI 0.03–0.22), I2 = 24%, *p*-value was 7%. There was a statistically significant difference in mortality between the two groups. In NIHK fetuses, the neonatal mortality with an OR of 0.17 (95% CI 0.12–0.25), I^2^ = 2%, *p*-value was 15%.

## Discussion

Fetal hyperechoic kidney (HEK) is a heterogeneous disorder in etiology (Huang et al., 2023). As we know, more than half of HEK are related to genetic factors. Fetal structural abnormality is the main indication for invasive prenatal genetic testing, which is traditionally performed by karyotyping and chromosomal microarray analysis (CMA). In recent years, next-generation sequencing technology, especially exome sequencing (ES), as a powerful tool for the diagnosis of Mendelian diseases, has been widely used in clinical practice, and can provide an additional diagnostic rate of 8.5%–11.6% for fetuses with abnormal ultrasound, normal karyotype and CMA results (Lord et al., 2019; Fu et al., 2022).

Based on the cohort study of hyperechoic kidney cases in recent years, we hope to find out the common etiologies and prognosis of hyperechoic kidney cases, evaluate the incidence and mortality of different causes, the efficacy of different detection methods for hyperechoic kidney cases, and explore the ultrasonic indicators that affect the prognosis of hyperechoic kidney cases. However, in recent years, the sample size in our cohort study was small, and the observable ultrasound findings were limited. Therefore, we included a retrospective cohort based on previous studies to increase the sample size and reduce the bias caused by insufficient samples. In addition, various ultrasound indicators affecting hyperechoic kidney cases, such as amniotic fluid volume, kidney size and CMD, were collected as far as possible. Another reason is that we wanted to verify the consistency with the results of the meta-analysis through a cohort study.

In our study, HEK included was classified as isolated and non-isolated and counted separately. In the cohort study, we found 25 cases of renal hyperechoic in recent years, accounting for about 10% of the cases of prenatal diagnosis of renal abnormalities and 0.6% of all cases of prenatal diagnosis of various indications. In IHK group, two fetuses were diagnosed with trisomy 21, which may seem unexpected, but they had different prenatal diagnostic indicators than ultrasound findings alone. Indications for non-invasive prenatal testing (NIPT) in NO. 19113 suggest a high risk of trisomy 21. Ultrasound images showed only bilateral hyperechoic kidneys. The prenatal diagnosis of NO. 19255 was indicative of advanced maternal age, the actual age was 40 years old, and ultrasound indicated bilateral hyperechoic kidneys of the fetus. Based on the indications, only karyotype and CNV-seq were tested in these two cases. However, hyperechoic kidney was found on ultrasound, we included these two cases in the IHK group. As we know, trisomy 21 syndrome may involve various systemic abnormalities, including some soft indicators, and of course may not be detected by ultrasound in the first and second trimester of pregnancy, perhaps as the pregnancy progresses, their ultrasound will show NIHK.

It is easy to find that the positive rate of CNV-seq in the NIHK group was higher than that in the IHK group. The positive rate of overall HEK fetal karyotype was 20%, and the abnormal detection rate of pathogenic CNVs was 32%, which was significantly higher than the detection rate of karyotype. The NIHK group also had a significantly higher TOP rate than the IHK group, with an overall TOP rate of 56%. However, due to the small sample size in our cohort, Fisher’s exact probability chi-square test showed that there was no significant difference in the positive rate and TOP rate of genetic testing between the IHK group and the NIHK group. Due to the combination of multiple structural abnormalities, the increased possibility of poor fetal prognosis judged by doctors, and the reasons of family members themselves, it is common for NIHK cases to refuse further WES and choose to terminate pregnancy, especially for families who have offspring and do not have the requirement of childbearing again. In the CNV-seq test results, we found that 6 fetuses carried variants of uncertain clinical significance (vous). At present, the correlation of these variants with ultrasonic manifestations and future pathogenicity is unknown. The risk of birth defects in fetuses without CNVs is currently 0.4% (Evans et al., 2016), so the risk of birth defects in fetuses with CNVs can be inferred to be no less than 0.4%. We recommend that the parents of the fetuses be tested for CNV-seq, indeed some of these variants are paternal or maternal, and there is no description of parental disease; However, some of the variants are still *de novo*. Although the current research shows that *de novo* variants are more likely to be pathogenic than hereditary variants, there is still no evidence to judge the prognosis of the fetus, and only regular ultrasound examination can be relied on to assess the survival status and prognosis of the fetus. Therefore, most families still choose to continue pregnancy. Because of the uncertainty about fetal outcomes has led a small number of families to refuse parental CNV-seq testing.

Trio-WES tests were performed for 5 cases in which a search for monogenic etiologies was deemed necessary by the physician. Case NO. 210040 prenatal diagnosis was indicated by advanced age of the pregnant woman (>35 years old), adverse pregnancy history (the last pregnancy was terminated due to pathogenic mutation of *PKHD1* gene in the fetus), and the fetal ultrasound of this pregnancy indicated bilateral hyperechoic kidneys of the fetus. Considering the possibility of monogenic etiologies of the fetus, Trio-WES testing was performed. The prenatal diagnosis indication of NO. 210086 was bilateral hyperechoic kidneys and oligohydramnios indicated by fetal ultrasound. Infantile polycystic kidney was considered, and WES examination was conducted to verify the ultrasound diagnosis. The fetal ultrasound of NO. 210323 indicated bilateral hyperechoic kidneys. Due to the gestational week exceeding 25 weeks, and the pregnant woman and her family members refused cordocentesis, considering the possibility of fetal monogenic cause, amniocentesis was performed and amniotic fluid CNV-seq and trio-WES were examined. The indication of NO. 220253 was bilateral hyperechoic kidneys indicated by fetal ultrasound. After communication with the pregnant woman, 10 mL of amniotic fluid was extracted during amniocentesis for preservation, and the chromosome karyotype and CNV-seq detection of amniotic fluid were performed first. However, CNV-seq results showed that no kidney related copy number variation was found except for X-linked ichthyosis pathogenic variation. Therefore, WES testing was performed on the preserved amniotic fluid. The fetal ultrasound of case NO. 170015 indicated multiple malformations, which was considered Meckel-Gruber syndrome. The family requested termination of the pregnancy and WES testing of the fetal cord of the induced labor was performed to verify the ultrasound diagnosis. In all 5 cases of WES, monogenic etiologies that could not be found by karyotypes and CNV-seq were found, especially *PKHD1* was more common in renal hyperechogenicity, followed by *PKD1* gene. However, WES results of case 4 (NO. 210086) in IHK group showed complex heterozygous variation of *PKHD1* gene, both vous, which were maternal and paternal, respectively. This can lead to PKD type 4 with or without polycystic liver disease (OMIM: 263200), although there seems to be some difficulty in making a definitive diagnosis. Therefore, in this case, the fetal ultrasound examination at 29 weeks gestation showed oligohydramnios (less than that at 24 weeks gestation), bilateral hyperechoic kidneys with enlarged volume, and small bladder, which considered infantile polycystic kidney. Although the ultrasound diagnosis was not completely consistent with the genetic diagnosis, the risk of poor prognosis of the fetus was comprehensively assessed, so we agreed with the parents’ decision to terminate the pregnancy based on the ultrasound diagnosis. For the 2 patients with 17q12 deletion syndrome whose CNVs were found, we did not perform WES testing again, but we speculated that *HNF1B* gene might be involved. These are common causes of hyperechoic kidney found in studies. Although some of our cases survived well within half a year to 1 year after delivery, some of them did not undergo genetic testing, and renal structural deterioration and even renal function decline may occur several years later, so these cases may need longer time follow-up and more frequent renal examination.

Unfortunately, we did not perform autopsy on fetuses obtained from termination of pregnancy, mainly because some pregnant women chose other hospitals for induced labor. The quantity of fetuses we obtained for induced labor was small, and ultrasound diagnosis of most fetuses was basically consistent with genetic diagnosis, so no autopsy was performed. Another reason is that the family cannot undergo an autopsy, although the individual ultrasound diagnosis is inconsistent with the genetic diagnosis, but before the termination of the pregnancy has shown ultrasonic manifestations such as oligohydramnios, and the ultrasound diagnosis predicts adverse fetal outcomes, the family has abandoned the autopsy, especially for those who have at least one child and no longer have the intention to have children.

The meta-analysis results showed that fetuses with hyperechoic kidney indicated by prenatal ultrasound had a risk of chromosomal and monogenic etiologies, especially in non-isolated cases. In cases of IHK, no positive results were found in 46 fetuses tested for karyotype. The positive rate of karyotype in NIHK was 22%, and that in HEK was 20%. This is consistent with the results in our cohort study. The positive rate of CMA was 32% in NIHK cases, previous studies (Nicolaides et al., 1992; Wapner et al., 2013) have shown that the incidence of chromosomal abnormalities is approximately 30% when multiple fetal abnormalities are detected, and is close to that in NIHK, however, this is lower than the 37% positive rate of the pathogenic variation of CNV-seq in our cohort study. The positive rate of CMA in IHK was 59%, which is similar to the results of a large Chinese cohort study (Su et al., 2022) but with a higher percentage, their finding was that hyperechoic kidney had the highest probability of diagnosis of pathogenic or probable pathogenic among all renal abnormalities detected by CMA (39.58%), IHK accounted for 44.44%, and NIHK was 25%. The rate of detection in the meta-analysis was higher than the 29% positive rate of CNV-seq testing in IHK cases in our series of studies. The reason for the large difference in results may be that the studies included in the meta-analysis, due to the limitations of the years and detection methods, not all cases were tested for CMA or CNV-seq; moreover, due to the small sample size tested, the positive ratio may eventually be high. Moreover, with the progress of pregnancy, some IHK cases may be transformed into NIHK by ultrasonography in the third trimester, but these cases were diagnosed as CMA positive in the second trimester, which may also be the reason for the high CMA positive rate in IHK cases.

Among the studies we retrieved and included, very few studies performed WES on HEK cases, and they mainly focused on NIHK cases, and some diagnoses were achieved by target gene sequencing. Since there are few studies on genetic testing of IHK cases and the detection method is not single, we analyzed the monogenic etiologies of IHK cases and found that the positive rate was 31%. Although a targeted gene sequencing article was excluded through sensitivity analysis, and the result was statistically significant, this single gene result may not fully explain the single gene etiology of IHK cases, targeted gene sequencing was performed in 3 of the 6 articles with single gene testing, and WES testing was performed in 8 cases in the remaining 3 articles, 5 of which were positive. As in our cohort study, all 5 WES tests were positive. Compared with WES, the diagnosis of IHK by targeted gene sequencing may be missed and the diagnosis rate may be reduced. In short, the quantity of IHK cases undergoing WES is too small, and more high-quality studies are needed to confirm it. Of course, it is not practical for all IHK cases undergo WES, but also according to the patient’s indications, especially for bilateral hyperechoic renal fetuses with negative karyotype and CMA or CNV-seq testing results. There may still be 31% of monogenic gene mutation. In all 28 cases of NIHK, the single gene results were positive, which seems easy to accept and expected.

Due to different years, detection methods are limited by testing technology, and some cases are diagnosed by clinical, ultrasound or autopsy cases. NIHK cases have large etiological heterogeneity due to the combination of abnormalities in each system, which we will not discuss. For the etiology of IHK cases, only 1 article (Li et al., 2020) reported aneuploidy, which could not truly reflect the proportion of aneuploidy in IHK. In addition, *HNF1B* variant is the most prevalent disease with 26%, which is contained in the 17q12 segment. In second place is ARPKD with a prevalence of 22%, ADPKD with 17%, Bardet-Biedl syndrome (BBS) and MCDK with 9% and 8%, respectively. There are other diseases that make up a large proportion of IHK, Such as *WTX* variant, renal tubular disease, nephroblastoma, autosomal dominant CAKUT (AD CAKUT), Type II Ivemark Syndrome, renal agenesis, Beckwith-Wiedemann syndrome (BWS). The good prognosis and disappearance of hyperechogenicity accounted for 23% of the cases.

In IHK cases, the subgroups with different amounts of amniotic fluid were subjected to sensitivity analysis, the mortality rate was 34% in oligohydramnios and 7% in the normal amniotic fluid group after sensitivity analysis, there was no deaths from IHK with polyhydramnios. Subgroup analysis showed that the mortality rate varied greatly with different amount of amniotic fluid and the results were statistically significant. Among NIHK cases, the mortality rate was 46% in the oligohydramnios group and 26% in the normal amniotic fluid group, and only one article reported the occurrence of fetal death in polyhydramnios, with a mortality rate of 13%. The large difference in mortality among the groups indicated that the amount of amniotic fluid was an important factor affecting fetal mortality.

The mortality rate in IHK was 36% in fetuses with enlarged kidneys and 7% in those with normal kidney size. No deaths were reported in fetuses with small kidney development. In the cases of NIHK, due to the limited number of relevant literature, the sensitivity analysis had little effect on the results of subgroup analysis, and the results were relatively stable. Therefore, the mortality rate of kidney enlargement was 55%, that of normal kidney was 22%, and that of relatively developed kidney was 3%. The mortality of NIHK was higher than that of IHK.

Termination (TOP) rates were 21% in IHK and 63% in NIHK. Although there was a large heterogeneity of TOP in both groups, the sensitivity analysis found that the results were relatively stable and meaningful, so the literature was not eliminated. In addition, the TOP probability is also affected by the results of prenatal diagnosis, which is partly related to the amniotic fluid volume and renal development changes suggested by ultrasound during pregnancy. Another part of the reason is that some pregnant women cannot accept further examination due to the limitation of previous diagnostic technology, family history of patients or some other subjective factors, and the attitude or legal requirements of hospitals in different countries on the behavior of pregnancy termination are not consistent, resulting in certain differences in the TOP rate in different regions. In addition, our assessment of the prognosis of HK is more focused on intrauterine death (IUD) and neonatal death (ND). In well-followed studies, the death of childhood kidney disease is also more significant for the long-term prognosis of IHK. No intrauterine deaths have been reported in IHK cases. Intrauterine mortality was 2% in NIHK cases. The neonatal mortality in IHK was 13%. NIHK has a large heterogeneity of neonatal mortality. After sensitivity analysis, 1study was excluded, in which 2 neonatal deaths were postoperative deaths of diaphragmatic hernia in the neonatal period. We were not sure whether the death was due to the disease itself or surgical complications, which led to unstable results, so it was no longer considered for inclusion, and the neonatal mortality was re-evaluated to be about 15%. 1study each reported childhood mortality in IHK and NIHK cases, with mortality rates of 10% and 3%, respectively. The childhood mortality rate of IHK is about three times higher than that of NIHK, which may not be the same as we expected. The main reason is that the number of literature is too small to conduct meta-analysis, and the number of childhood deaths reported by only one article is not enough to describe the real mortality rate. Secondly, we can also understand that NIHK patients with poor prognosis died before childhood due to their own combined abnormalities of other systems, both intrarenal and extrarenal. In addition, the follow-up of childhood deaths requires a long period of time, and different duration of follow-up also affects the long-term mortality results. Therefore, we may need more articles with long-term follow-up to understand the long-term survival and mortality of this disease. Not all survivors are healthy. 22% of IHK survivors are still likely to face hypertension, diabetes, renal failure, dialysis, kidney transplantation, etc., compared with 29% of NIHK survivors.

Fetuses with poor or absent CMD have a higher mortality rate of 65%. Cortico-medullary differentiation, a decrease in medullary and cortical thickness, is a hallmark of renal dysplasia (Devriendt et al., 2013), as a result, fetal mortality is higher, including family abandonment and death during pregnancy.

This meta-analysis comprehensively covered the etiology of IHK, the incidence of HEK monogenic etiologies, mortality, and prognostic healthy survival, disease survival, and prognostic survival factors. The main limitation of the current study is that most of the studies included were retrospective designs and some of the outcomes evaluated were reported only in a limited proportion of the included studies. In addition, as not all cases in the included studies were genetically tested, and not all were routinely measured kidney length, CMD, were only tested or measured when abnormalities were apparent, there may be selection bias. Nonetheless, the available data are sufficient to draw the conclusions.

## Conclusion

The positive rate of karyotype was 20% in HEK and 22% in NIHK. The positive rate of CMA was 32% in NIHK and 59% in IHK. The positive rate of IHK monogenic etiologies was 31%. The most common etiology of IHK is the *HNF1B* variant. Oligohydramnios, renal enlargement and CMD with two or more items increase the possibility of poor prognosis. We currently recommend a detailed ultrasound scan of the HEK fetus, measuring kidney length, CMD in addition to the routine measurement of amniotic fluid, and periodically rechecking for changes. Interventional prenatal diagnosis is performed, including chromosome karyotype and CMA or CNV-seq, and WES testing is feasible when necessary, especially if the karyotype and CMA results are negative, do not ignore the possibility of monogenic etiologies Combined with various indicators, comprehensive evaluation of fetal prognosis, avoid unnecessary termination of pregnancy, and also reduce the birth of defects to the greatest extent.

## Data Availability

The original contributions presented in the study are included in the article/Supplementary Material, further inquiries can be directed to the corresponding author.

## Supplementary material

SUPPLEMENTARY FIGURE S1 Subgroup analysis of different amniotic fluid mortality rates in IHK.

SUPPLEMENTARY FIGURE S2 Subgroup analysis of different amniotic fluid mortality rates in NIHK.

SUPPLEMENTARY FIGURE S3 Subgroup analysis of different kidney size mortality rates in IHK.

SUPPLEMENTARY FIGURE S4 Subgroup analysis of different kidney size mortality rates in NIHK.

SUPPLEMENTARY FIGURE S5 Sensitivity analysis of positive rate of IHK monogenic etiologies.

SUPPLEMENTARY FIGURE S6 Sensitivity analysis for subgroup analysis of IHK mortality by different amount of amniotic fluid.

SUPPLEMENTARY FIGURE S7 Sensitivity analysis of neonatal mortality heterogeneity in NIHK.
