# Supplementary material for: Fetal hyperechoic kidney cohort study and a meta-analysis
Source: Front Genet. 2023 Aug 17;14:1237912. doi: 10.3389/fgene.2023.1237912 (PMC10469696; doi:10.3389/fgene.2023.1237912)
Supplement: Supplementary file 1 [file DataSheet2.PDF]

Search number	Query
9	(((((("fetus"[MeSH Terms]) AND (1945/1/1:2023/2/28[pdat]) AND (1945/1/1:2023/2/28[pdat])) OR (("Fetuses"[Title/Abstract] OR "fetal structure*"[Title/Abstract] OR "structure fetal"[Title/Abstract] OR "mummified fetus"[Title/Abstract] OR "fetus mummified"[Title/Abstract] OR "retained fetus"[Title/Abstract] OR "fetus retained"[Title/Abstract] OR "fetal tissue*"[Title/Abstract] OR "tissue fetal"[Title/Abstract]) AND (1945/1/1:2023/2/28[pdat]) AND (1945/1/1:2023/2/28[pdat]))) OR (("antenatal"[Title/Abstract] OR "prenatal"[Title/Abstract]) AND (1945/1/1:2023/2/28[pdat]) AND (1945/1/1:2023/2/28[pdat])) AND (1945/1/1:2023/2/28[pdat])) AND (("Kidney"[MeSH Terms] OR "kidneys"[Title/Abstract]) AND (1945/1/1:2023/2/28[pdat]) AND (1945/1/1:2023/2/28[pdat]))) AND (("hyperecho*"[Title/Abstract]) AND (1945/1/1:2023/2/28[pdat]) AND (1945/1/1:2023/2/28[pdat])) AND (1945/1/1:2023/2/28[pdat])) AND (("cohort studies"[MeSH Terms] OR "case-control studies"[MeSH Terms] OR "comparative study"[Publication Type] OR "risk factors"[MeSH Terms] OR "cohort"[Text Word] OR "compared"[Text Word] OR "groups"[Text Word] OR "case control"[Text Word] OR "multivariate"[Text Word]) AND (1945/1/1:2023/2/28[pdat]) AND (1945/1/1:2023/2/28[pdat]))
8	("cohort studies"[MeSH Terms] OR "case-control studies"[MeSH Terms] OR "comparative study"[Publication Type] OR "risk factors"[MeSH Terms] OR "cohort"[Text Word] OR "compared"[Text Word] OR "groups"[Text Word] OR "case control"[Text Word] OR "multivariate"[Text Word]) AND (1945/1/1:2023/2/28[pdat])

7	((((("fetus"[MeSH Terms]) AND (1945/1/1:2023/2/28[pdat]) AND (1945/1/1:2023/2/28[pdat])) OR (("Fetuses"[Title/Abstract] OR "fetal structure*"[Title/Abstract] OR "structure fetal"[Title/Abstract] OR "mummified fetus"[Title/Abstract] OR "fetus mummified"[Title/Abstract] OR "retained fetus"[Title/Abstract] OR "fetus retained"[Title/Abstract] OR "fetal tissue*"[Title/Abstract] OR "tissue fetal"[Title/Abstract]) AND (1945/1/1:2023/2/28[pdat]) AND (1945/1/1:2023/2/28[pdat]))) OR (("antenatal"[Title/Abstract] OR "prenatal"[Title/Abstract]) AND (1945/1/1:2023/2/28[pdat]) AND (1945/1/1:2023/2/28[pdat])) AND (1945/1/1:2023/2/28[pdat])) AND (("Kidney"[MeSH Terms] OR "kidneys"[Title/Abstract]) AND (1945/1/1:2023/2/28[pdat]) AND (1945/1/1:2023/2/28[pdat]))) AND (("hyperecho*"[Title/Abstract]) AND (1945/1/1:2023/2/28[pdat]) AND (1945/1/1:2023/2/28[pdat]))
6	("hyperecho*"[Title/Abstract]) AND (1945/1/1:2023/2/28[pdat])
5	("Kidney"[MeSH Terms] OR "kidneys"[Title/Abstract]) AND (1945/1/1:2023/2/28[pdat])
4	((("fetus"[MeSH Terms]) AND (1945/1/1:2023/2/28[pdat]) AND (1945/1/1:2023/2/28[pdat])) OR (("Fetuses"[Title/Abstract] OR "fetal structure*"[Title/Abstract] OR "structure fetal"[Title/Abstract] OR "mummified fetus"[Title/Abstract] OR "fetus mummified"[Title/Abstract] OR "retained fetus"[Title/Abstract] OR "fetus retained"[Title/Abstract] OR "fetal tissue*"[Title/Abstract] OR "tissue fetal"[Title/Abstract]) AND (1945/1/1:2023/2/28[pdat]) AND (1945/1/1:2023/2/28[pdat]))) OR (("antenatal"[Title/Abstract] OR "prenatal"[Title/Abstract]) AND (1945/1/1:2023/2/28[pdat]) AND (1945/1/1:2023/2/28[pdat]))
3	("antenatal"[Title/Abstract] OR "prenatal"[Title/Abstract]) AND (1945/1/1:2023/2/28[pdat])
2	("Fetuses"[Title/Abstract] OR "fetal structure*"[Title/Abstract] OR "structure fetal"[Title/Abstract] OR "mummified fetus"[Title/Abstract] OR "fetus mummified"[Title/Abstract] OR "retained fetus"[Title/Abstract] OR "fetus retained"[Title/Abstract] OR "fetal tissue*"[Title/Abstract] OR "tissue fetal"[Title/Abstract]) AND (1945/1/1:2023/2/28[pdat])

1	("fetus"[MeSH Terms]) AND (1945/1/1:2023/2/28[pdat])

Sort By   Filters

Search Details	Results
(("fetus"[MeSH Terms] AND 1945/01/01:2023/02/28[Date - Publication] AND 1945/01/01:2023/02/28[Date - Publication]) OR (("Fetuses"[Title/Abstract] OR "fetal structure*"[Title/Abstract] OR "structure fetal"[Title/Abstract] OR "mummified fetus"[Title/Abstract] OR "fetus mummified"[Title/Abstract] OR "retained fetus"[Title/Abstract] OR "fetus retained"[Title/Abstract] OR "fetal tissue*"[Title/Abstract] OR "tissue fetal"[Title/Abstract]) AND 1945/01/01:2023/02/28[Date - Publication] AND 1945/01/01:2023/02/28[Date - Publication]) OR (("antenatal"[Title/Abstract] OR "prenatal"[Title/Abstract]) AND 1945/01/01:2023/02/28[Date - Publication] AND 1945/01/01:2023/02/28[Date - Publication])) AND 1945/01/01:2023/02/28[Date - Publication] AND (("Kidney"[MeSH Terms] OR "kidneys"[Title/Abstract]) AND 1945/01/01:2023/02/28[Date - Publication] AND 1945/01/01:2023/02/28[Date - Publication]) AND ("hyperecho*"[Title/Abstract] AND 1945/01/01:2023/02/28[Date - Publication] AND 1945/01/01:2023/02/28[Date - Publication]) AND 1945/01/01:2023/02/28[Date - Publication] AND (("cohort studies"[MeSH Terms] OR "case-control studies"[MeSH Terms] OR "comparative study"[Publication Type] OR "risk factors"[MeSH Terms] OR "cohort"[Text Word] OR "compared"[Text Word] OR "groups"[Text Word] OR "case control"[Text Word] OR "multivariate"[Text Word]) AND 1945/01/01:2023/02/28[Date - Publication] AND 1945/01/01:2023/02/28[Date - Publication])	37
("cohort studies"[MeSH Terms] OR "case-control studies"[MeSH Terms] OR "comparative study"[Publication Type] OR "risk factors"[MeSH Terms] OR "cohort"[Text Word] OR "compared"[Text Word] OR "groups"[Text Word] OR "case control"[Text Word] OR "multivariate"[Text Word]) AND 1945/01/01:2023/02/28[Date - Publication]	9,343,079

(("fetus"[MeSH Terms] AND 1945/01/01:2023/02/28[Date - Publication] AND 1945/01/01:2023/02/28[Date - Publication]) OR (("Fetuses"[Title/Abstract] OR "fetal structure*"[Title/Abstract] OR "structure fetal"[Title/Abstract] OR "mummified fetus"[Title/Abstract] OR "fetus mummified"[Title/Abstract] OR "retained fetus"[Title/Abstract] OR "fetus retained"[Title/Abstract] OR "fetal tissue*"[Title/Abstract] OR "tissue fetal"[Title/Abstract]) AND 1945/01/01:2023/02/28[Date - Publication] AND 1945/01/01:2023/02/28[Date - Publication]) OR (("antenatal"[Title/Abstract] OR "prenatal"[Title/Abstract]) AND 1945/01/01:2023/02/28[Date - Publication] AND 1945/01/01:2023/02/28[Date - Publication])) AND 1945/01/01:2023/02/28[Date - Publication] AND (("Kidney"[MeSH Terms] OR "kidneys"[Title/Abstract]) AND 1945/01/01:2023/02/28[Date - Publication] AND 1945/01/01:2023/02/28[Date - Publication]) AND ("hyperecho*"[Title/Abstract] AND 1945/01/01:2023/02/28[Date - Publication] AND 1945/01/01:2023/02/28[Date - Publication])	115
"hyperecho*"[Title/Abstract] AND 1945/01/01:2023/02/28[Date - Publication]	5,404
("Kidney"[MeSH Terms] OR "kidneys"[Title/Abstract]) AND 1945/01/01:2023/02/28[Date - Publication]	422,303
("fetus"[MeSH Terms] AND 1945/01/01:2023/02/28[Date - Publication] AND 1945/01/01:2023/02/28[Date - Publication]) OR (("Fetuses"[Title/Abstract] OR "fetal structure*"[Title/Abstract] OR "structure fetal"[Title/Abstract] OR "mummified fetus"[Title/Abstract] OR "fetus mummified"[Title/Abstract] OR "retained fetus"[Title/Abstract] OR "fetus retained"[Title/Abstract] OR "fetal tissue*"[Title/Abstract] OR "tissue fetal"[Title/Abstract]) AND 1945/01/01:2023/02/28[Date - Publication] AND 1945/01/01:2023/02/28[Date - Publication]) OR (("antenatal"[Title/Abstract] OR "prenatal"[Title/Abstract]) AND 1945/01/01:2023/02/28[Date - Publication] AND 1945/01/01:2023/02/28[Date - Publication])	331,895
("antenatal"[Title/Abstract] OR "prenatal"[Title/Abstract]) AND 1945/01/01:2023/02/28[Date - Publication]	152,549
("Fetuses"[Title/Abstract] OR "fetal structure*"[Title/Abstract] OR "structure fetal"[Title/Abstract] OR "mummified fetus"[Title/Abstract] OR "fetus mummified"[Title/Abstract] OR "retained fetus"[Title/Abstract] OR "fetus retained"[Title/Abstract] OR "fetal tissue*"[Title/Abstract] OR "tissue fetal"[Title/Abstract]) AND 1945/01/01:2023/02/28[Date - Publication]	59,965

"fetus"[MeSH Terms] AND 1945/01/01:2023/02/28[Date - Publication]	166,598

Time

2:25:54

2:22:27

2:21:11

2:18:04

2:17:12

2:16:07

2:13:10

2:11:17

2:10:05
