# Supplementary figures and images for: Fetal hyperechoic kidney cohort study and a meta-analysis
Source: Front Genet. 2023 Aug 17;14:1237912. doi: 10.3389/fgene.2023.1237912 (PMC10469696; doi:10.3389/fgene.2023.1237912)

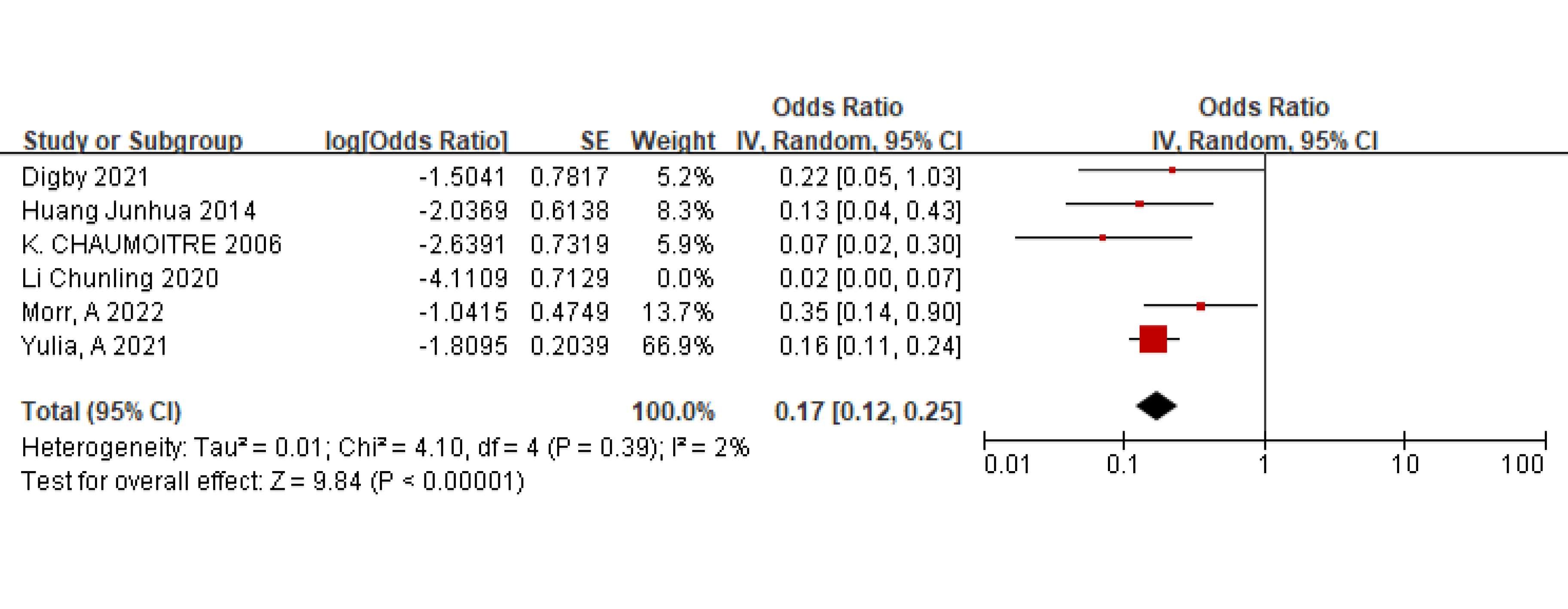

Supplement: Supplementary file 2 [file Image6.TIF]

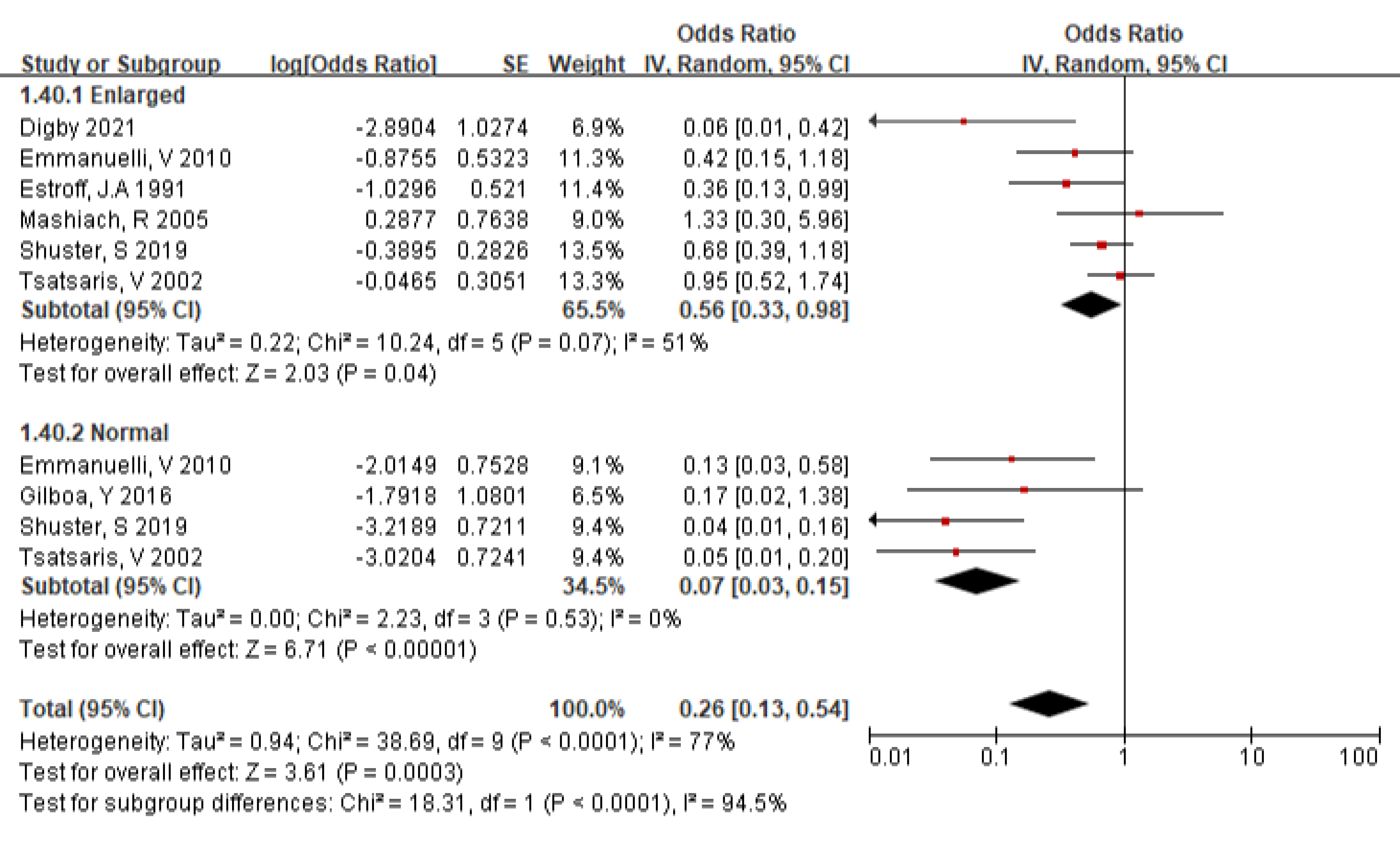

Supplement: Supplementary file 3 [file Image3.TIF]

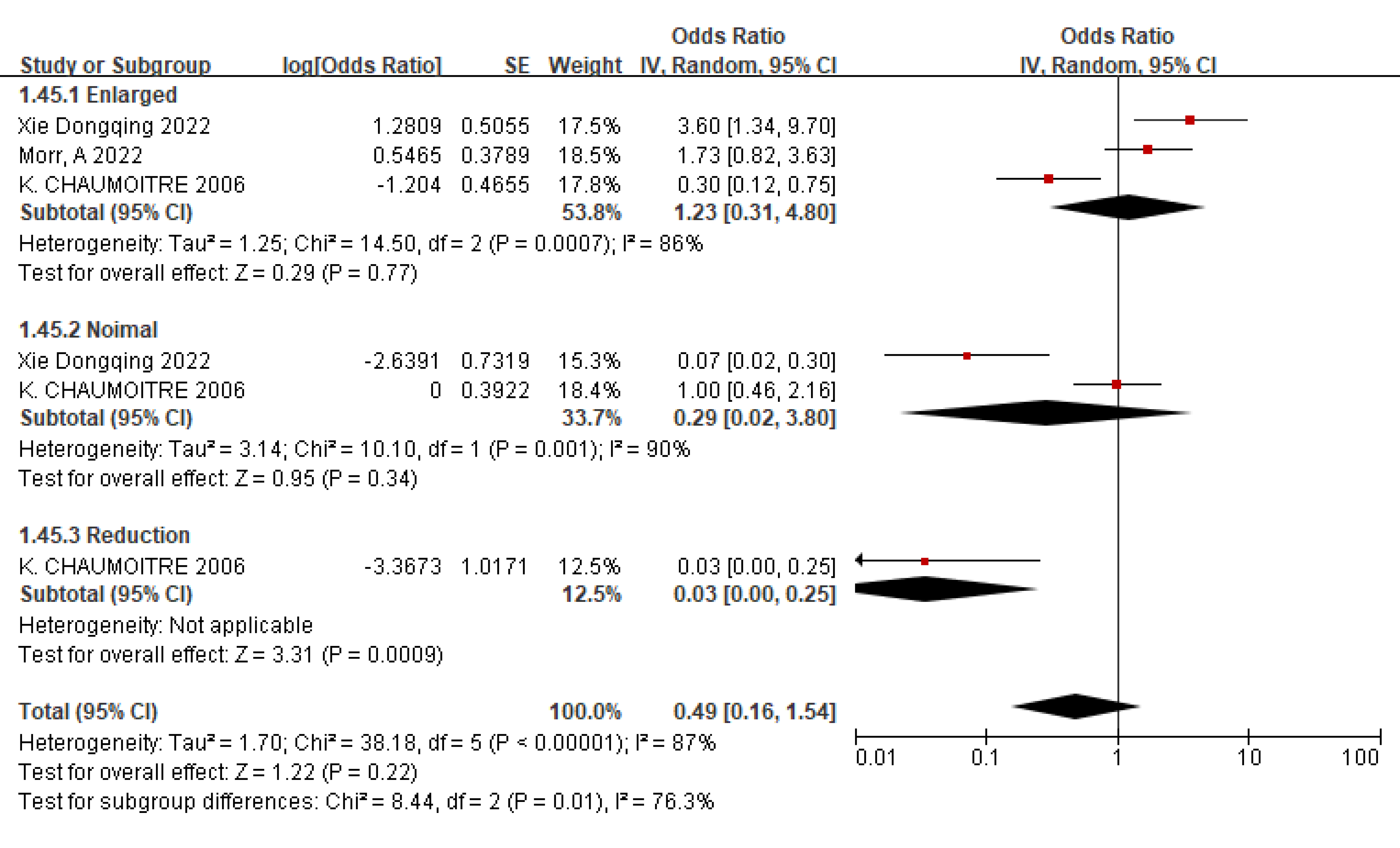

Supplement: Supplementary file 4 [file Image4.TIF]

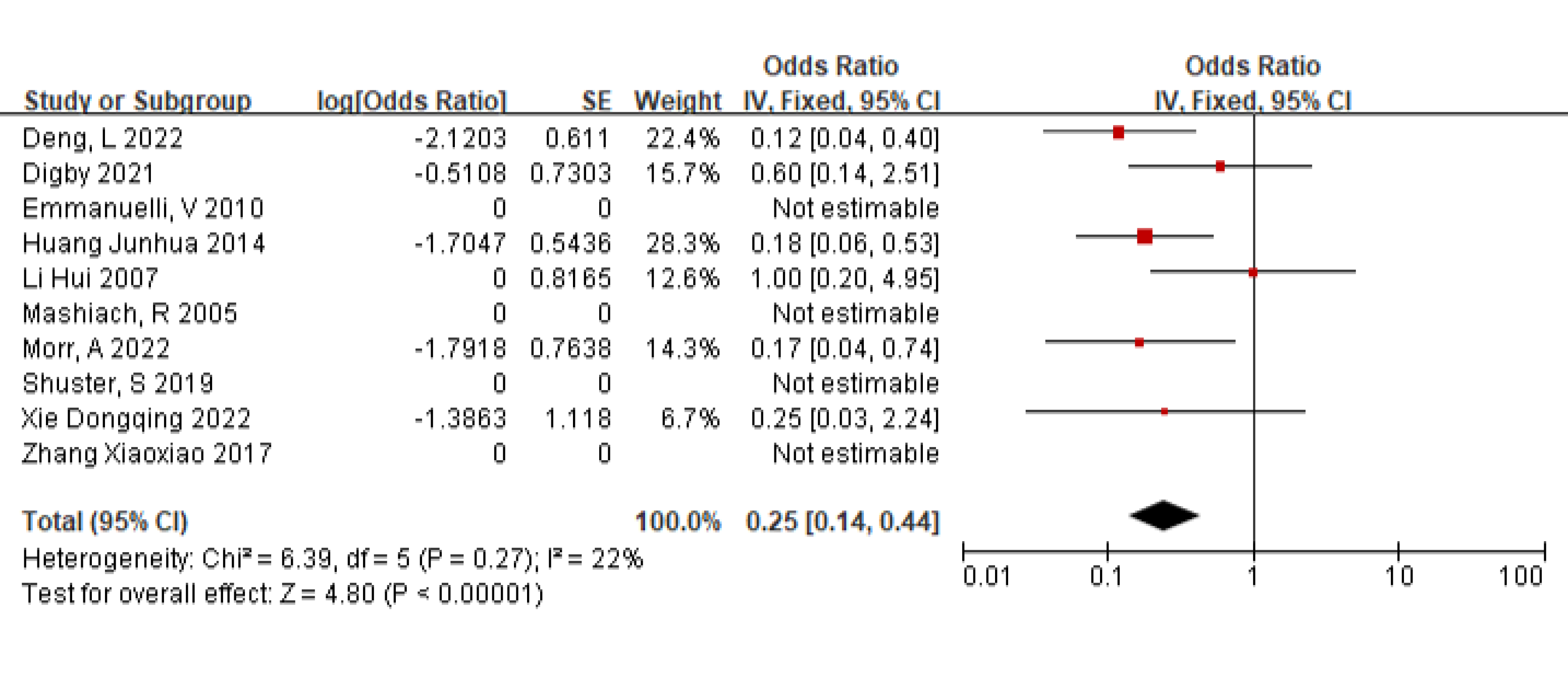

Supplement: Supplementary file 5 [file Image9.TIF]

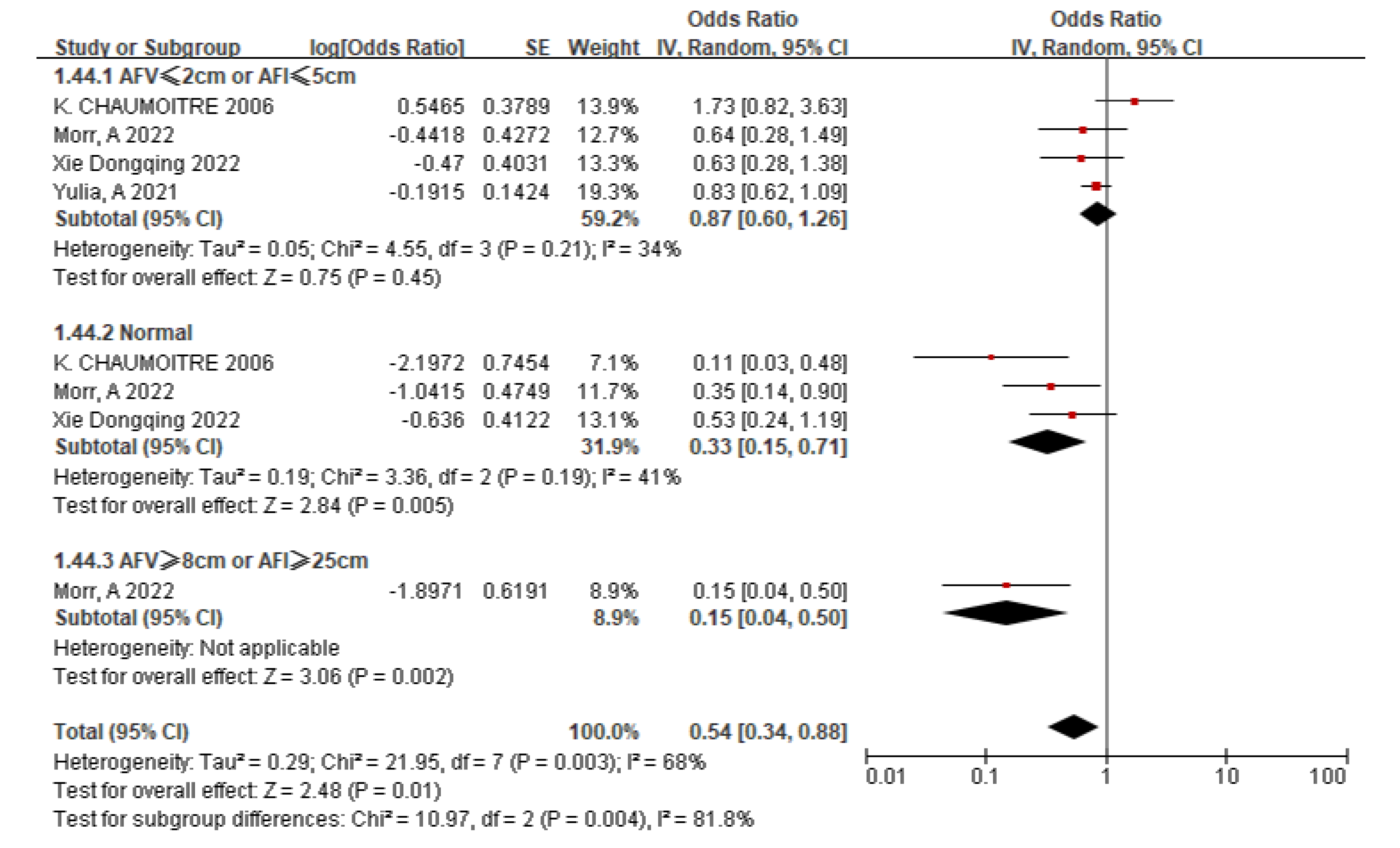

Supplement: Supplementary file 6 [file Image2.TIF]

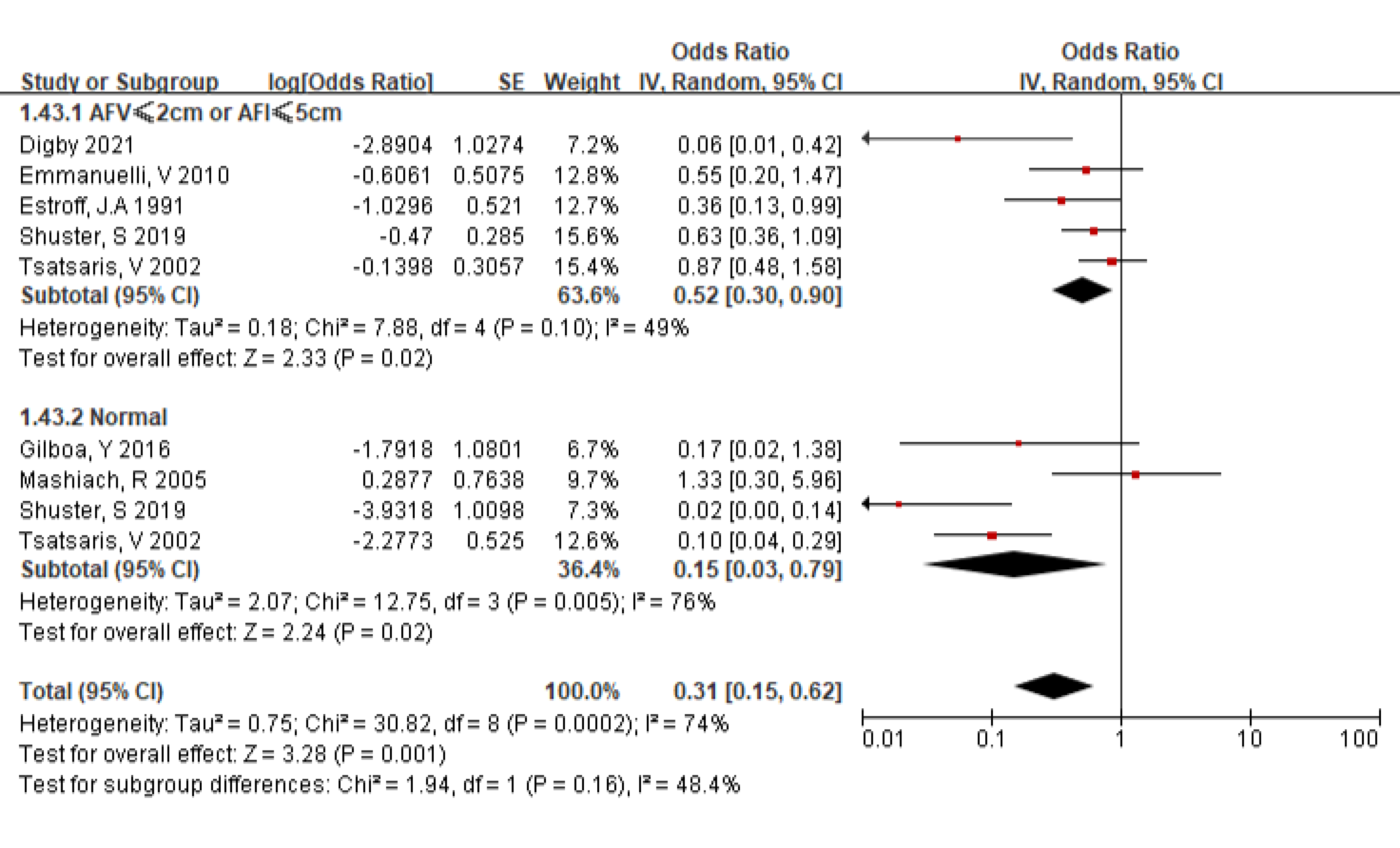

Supplement: Supplementary file 7 [file Image1.TIF]

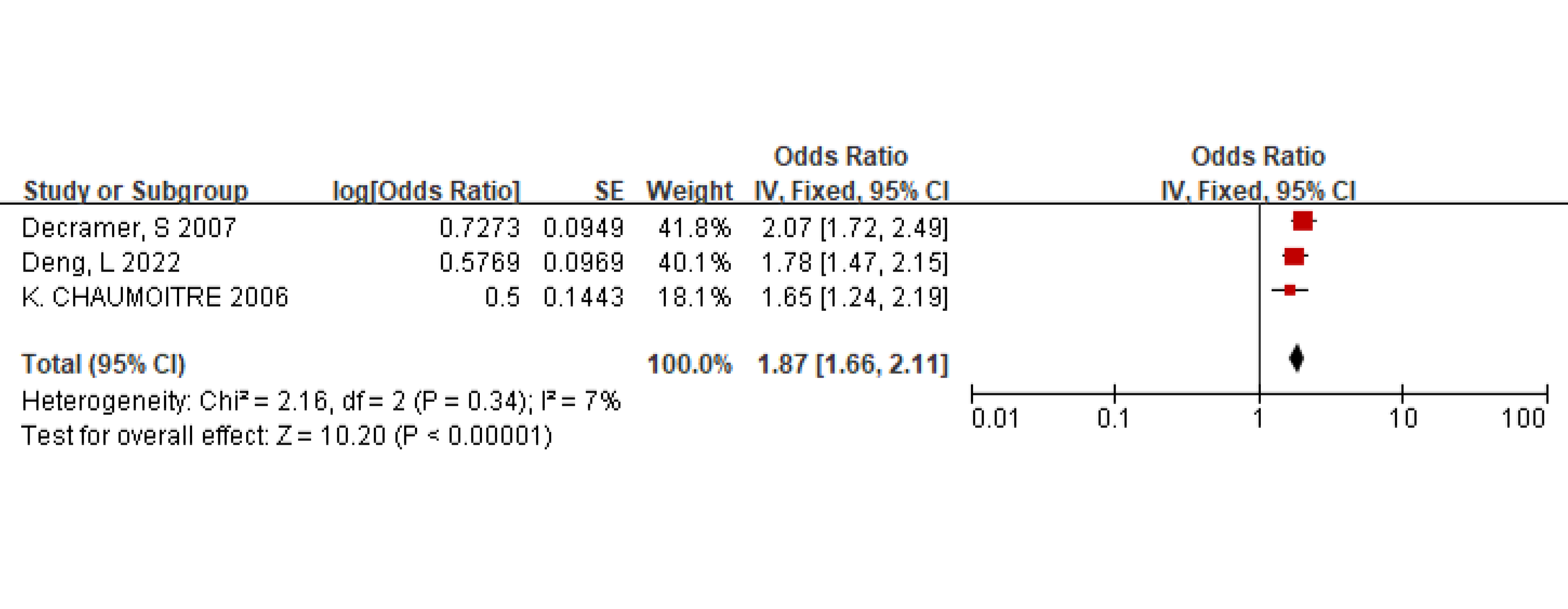

Supplement: Supplementary file 8 [file Image7.TIF]

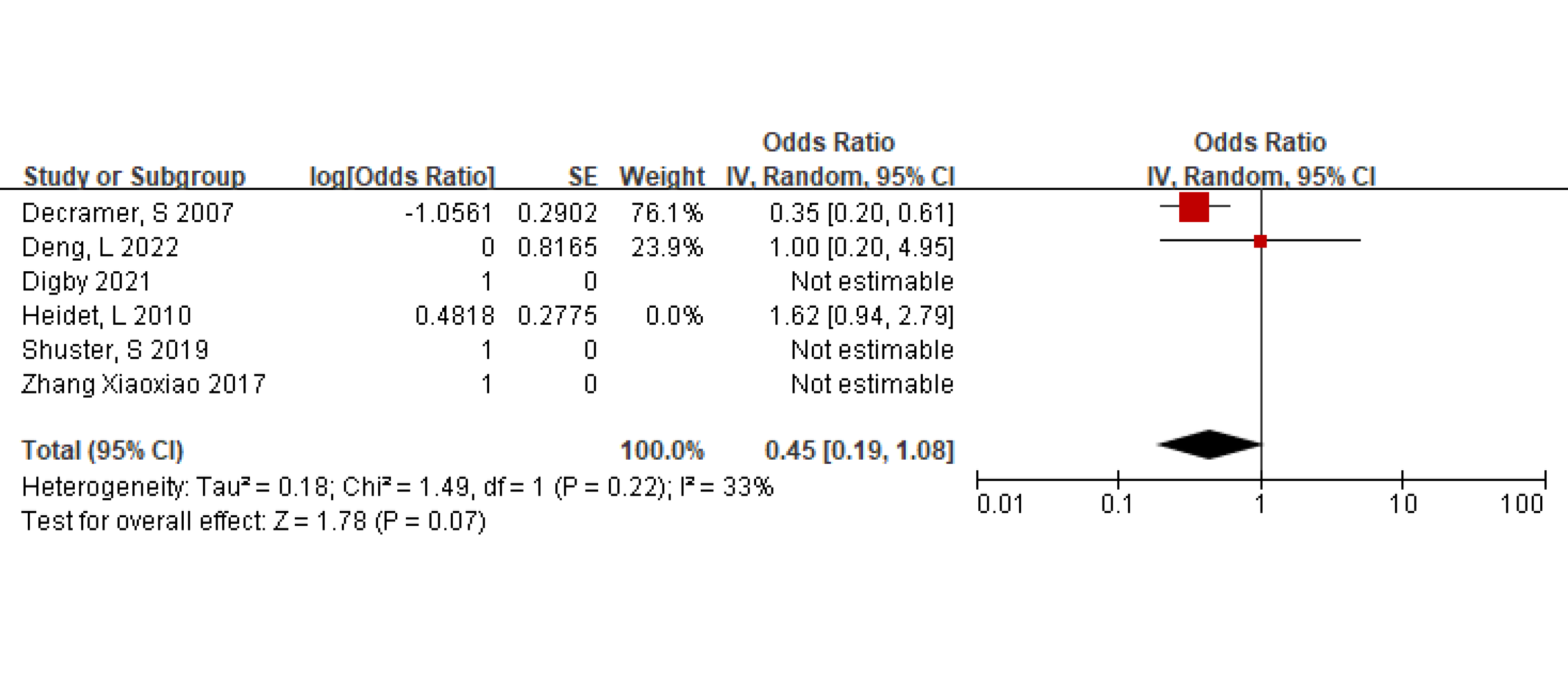

Supplement: Supplementary file 9 [file Image8.TIF]

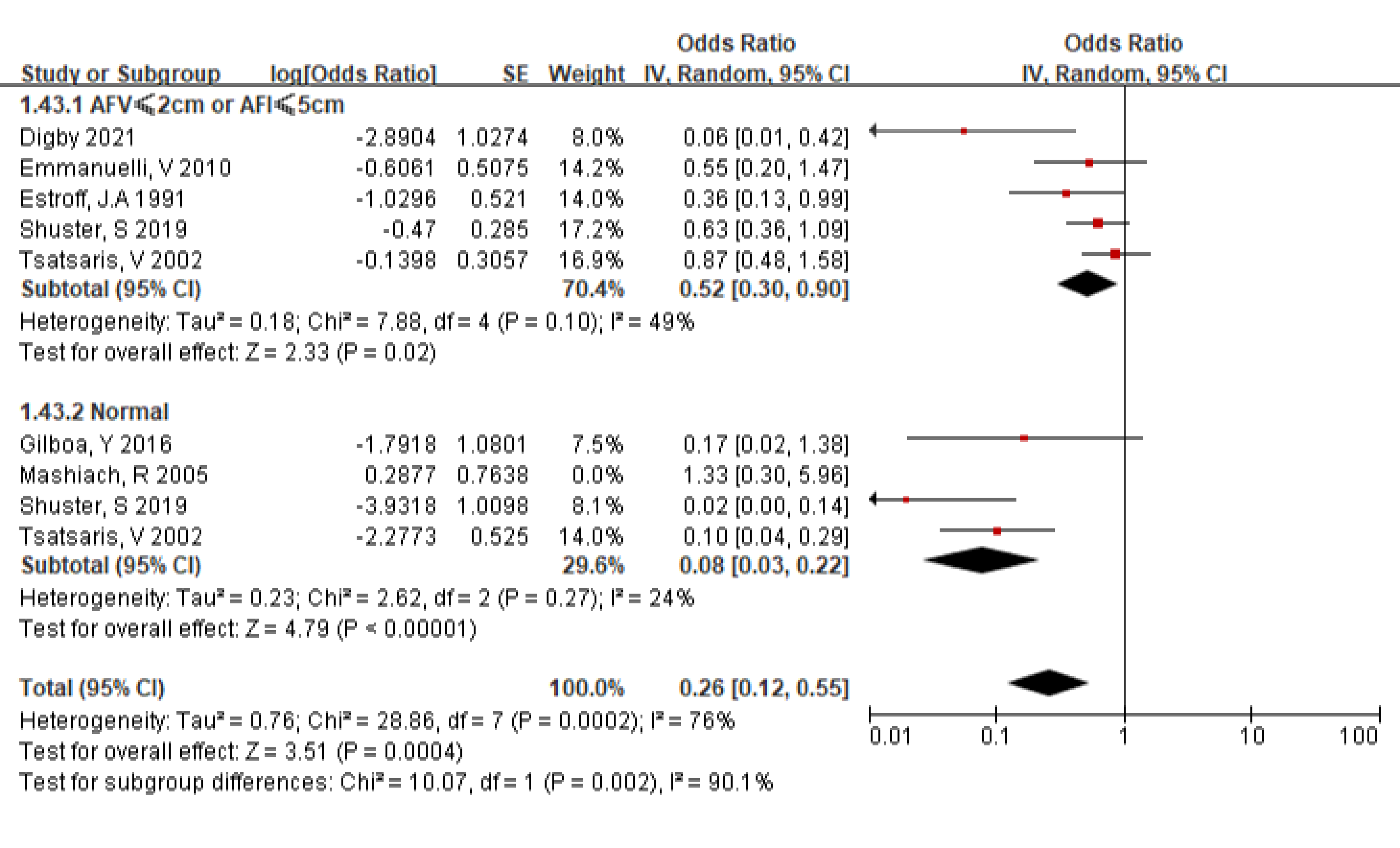

Supplement: Supplementary file 10 [file Image5.TIF]
